# Two-plex *in vivo* molecular imaging in the second near-infrared window for immunotherapeutic response

**DOI:** 10.7150/thno.108880

**Published:** 2025-03-19

**Authors:** Yupeng Sun, Rui Li, Yike Cai, Yan Liu, Peiyuan Wang, Ming Wu, Xiaolong Zhang, Naishun Liao, Cuilin Zhang, Aixian Zheng, Haipo Xu, Rui Zeng, Yongyi Zeng, Xiaolong Liu

**Affiliations:** 1 The United Innovation of Mengchao Hepatobiliary Technology Key Laboratory of Fujian Province, Mengchao Hepatobiliary Hospital of Fujian Medical University, Fuzhou 350025, P. R. China.; 2 Mengchao Med-X Center, Fuzhou University, Fuzhou 350116, P. R. China.; 3 College of Biological Science and Engineering, Fuzhou University, Fuzhou, 350116, P. R. China.; 4 Liver Disease Center, the First Affiliated Hospital of Fujian Medical University, Fuzhou 350005, P. R. China.; 5 CAS Key Laboratory of Design and Assembly of Functional Nanostructures, Fujian Institute of Research on the Structure of Matter, Chinese Academy of Sciences, Fuzhou 350002, P. R. China.

**Keywords:** *in vivo* imaging, second near-infrared window (NIR-II), two-plex molecular imaging, tumor-infiltrating CD8^+^ T cells, programmed death-1 (PD1), immunotherapeutic response

## Abstract

Tumor-infiltrating CD8^+^ T cells and programmed death-1 (PD1) levels are critical indicators for tumor immunophenotyping and therapeutic decision-making. Noninvasive optical imaging in the second near infrared window (NIR-II) is particularly well-suited for investigating the biological processes within tumors in live mammals, thanks to its deep-tissue penetration and superior spatiotemporal resolution. However, *in vivo* NIR-II imaging has primarily been restricted to a single probe at a time.

**Methods:** Herein, we developed a two-plex NIR-II molecular imaging method utilizing the non-overlapping fluorescence emission of indocyanine green (ICG) in the NIR-IIa window (1000-1200 nm) and PbS/CdS core-shell quantum dots (QDs) in the NIR-IIb window (1500-1700 nm). By integrating PD1 aptamer-labeled ICG (ICG-Apt-PD1, targeting PD1) and CD8 aptamer-labeled QDs (QDs@Apt-CD8, targeting CD8^+^ T cells), our two-plex NIR-II molecular imaging enabled simultaneous and noninvasive monitoring of the number of CD8^+^ T cells and PD1 levels in tumors.

**Results:** QDs@Apt-CD8 demonstrated the excellent ability for *in vivo* imaging of tumor infiltrating CD8^+^ T cells, owing to its strong NIR-IIb luminescence and the high selectivity and specificity. This two-plex *in vivo* molecular imaging allowed for dynamic monitoring for PD1 levels and the number of CD8^+^ T cells in tumors. We observed the heterogeneous bio-distributions of PD1 and CD8^+^ T cells across different tumor types and revealed the tumor immunophenotypes. Moreover, our findings indicated that the low PD1 and high CD8^+^ T cells levels in tumors predicted a better anti-tumor effect.

**Conclusions:** Such *in vivo* noninvasive NIR-II molecular imaging would complement *ex vivo* biopsy-based diagnostic techniques, and it could contribute to developing an *in vivo* tumor immune-scoring algorithm to offer a more precise prediction for immunotherapeutic response.

## Introduction

Immunotherapy harnesses the power of the body's immune system to eliminate cancer cells, offering a promising alternative to traditional methods such as surgery, radiation therapy and chemotherapy 1. Notable progresses have been made in developing the immune checkpoint blockades (ICB), which have demonstrated significant clinical efficacy across various cancers. Unfortunately, the response rates to ICB remain suboptimal, largely due to the tumor immune microenvironment (TIME), which plays a critical role in determining the therapeutic effectiveness 2, 3. Tumor-infiltrating CD8^+^ T cells, a key component of TIME, could recognize and destroy cancer cells by activating or eliciting the adaptive immune responses 4. However, tumor cells also could gradually establish a complex and dynamic immune-suppressive microenvironment during tumor evolution, leading to a "exhausted" state in CD8^+^ T cells 5. Generally, CD8^+^ T cells in the "exhausted" immune state exhibited a high level of PD1, which is regarded as a key biomarker for T cell exhaustion, and its expression level influences the efficacy of immunotherapy and tumor progression 6, 7. Therefore, it`s vital to understand the intricate landscape of CD8^+^ T cells and PD1 levels in tumors for effectively guiding ICB and developing next-generation immunotherapies.

In general, flow cytometry (FCM) and RNA sequencing have been employed to analyze the subsets of immune cells and their immune status 8, 9, however, it needs to prepare single-cell suspensions, which removes cells from their native immune microenvironment, resulting in the loss of spatial-position information and the altered immune status. Although invasive surgery and installing transparent optical windows have been used for *in vivo* molecular imaging of the tumor microenvironment, which tolerated complex procedures and undesired stimulation, potentially resulting in the inaccurate assessments of the immunotherapeutic response 10, 11. Non-invasive *in vivo* fluorescence imaging can provide direct insights into the cellular or molecular behaviors of living animals 12, and holds promise for dynamically monitoring of the tumor-infiltrating CD8^+^ T cells and PD1 levels. Nevertheless, it still remains a challenge due to the hindrance and scattering of fluorescent signals caused by body tissues or blood 13, 14. NIR-II fluorescence imaging offers deeper tissue penetration with higher clarity, due to reduced photon scattering and diminished tissue autofluorescence 15-18.

So far, the NIR-II fluorescence probes, including small molecules 19, 20, gold nanoclusters 21, 22, polymer-integrated organic dyes 23-25, quantum dots (QDs) 26, 27, and rare-earth nanoparticles (RENPs) 28, 29 have been used for *in vivo* imaging, tracking the specific biological molecule, and even clinical applications such as non-invasive diagnostics and image-guided surgery. However, *in vivo* NIR-II imaging has primarily been restricted to a single probe at a time 30, 31, making it difficult to investigate live biological and physiological processes involving two or more important biomolecules simultaneously.

**Scheme 1:**
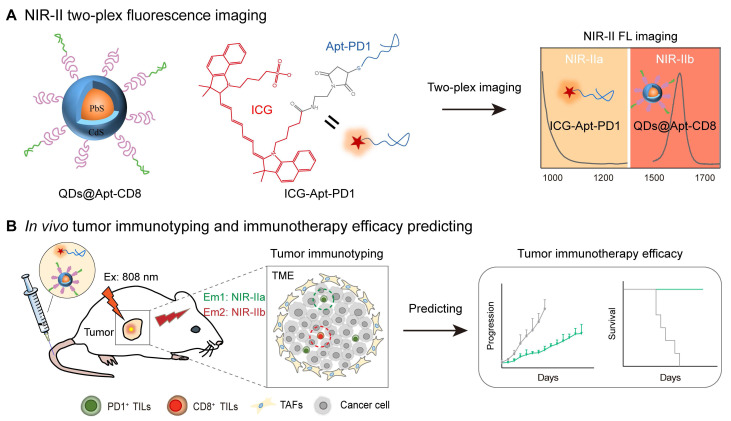
Illustration of *in vivo* two-plex molecular imaging in the NIR-II window for tumor-infiltrating CD8^+^ T cells and PD1 markers. (A) Fabrication of two-plex NIR-II fluorescence imaging method utilizing the non-overlap emission of indocyanine green (ICG) in the NIR-IIa window and PbS/CdS QDs in the NIR-IIb window. (B) Evaluation of tumor immunophenotypes and prediction of tumor immunotherapy efficacy through *in vivo* imaging of the number of CD8^+^ T cells and PD1 levels in tumors.

Herein, we employed indocyanine green (ICG) and PbS/CdS core-shell quantum dots (QDs) with the non-overlapping fluorescence emission in the NIR-II window to develop a two-plex NIR-II fluorescence imaging strategy (Scheme 1). ICG is a clinically approved near-infrared tracer known for its excellent safety profile and tailing fluorescence emission in the NIR-IIa window (1000-1200 nm) 32. On the other hand, PbS/CdS core-shell QDs are nanoscale semiconductor crystals with a CdS shell that provides chemical passivation while retaining the luminescence of the PbS core, emitting intense fluorescence in the NIR-IIb window (1500-1700 nm) 33. Here, we selected aptamers as the targeting ligands to specifically recognize CD8^+^ T cells and PD1 markers 34-36. Two-plex NIR-II molecular imaging using PD1 aptamer-labeled ICG (ICG-Apt-PD1, targeting PD1) and CD8 aptamer-labeled QDs (QDs@Apt-CD8, targeting CD8^+^ T cells) enabled noninvasive *in vivo* monitoring of the number of CD8^+^ T cells and PD1 levels within tumors, allowing for the evaluation of tumor immunophenotypes and prediction of the therapeutic efficacy of tumor immunotherapy.

## Results and Discussion

### Synthesis and characterization of QDs@Apt-CD8 nanoprobe

**Figure 1:**
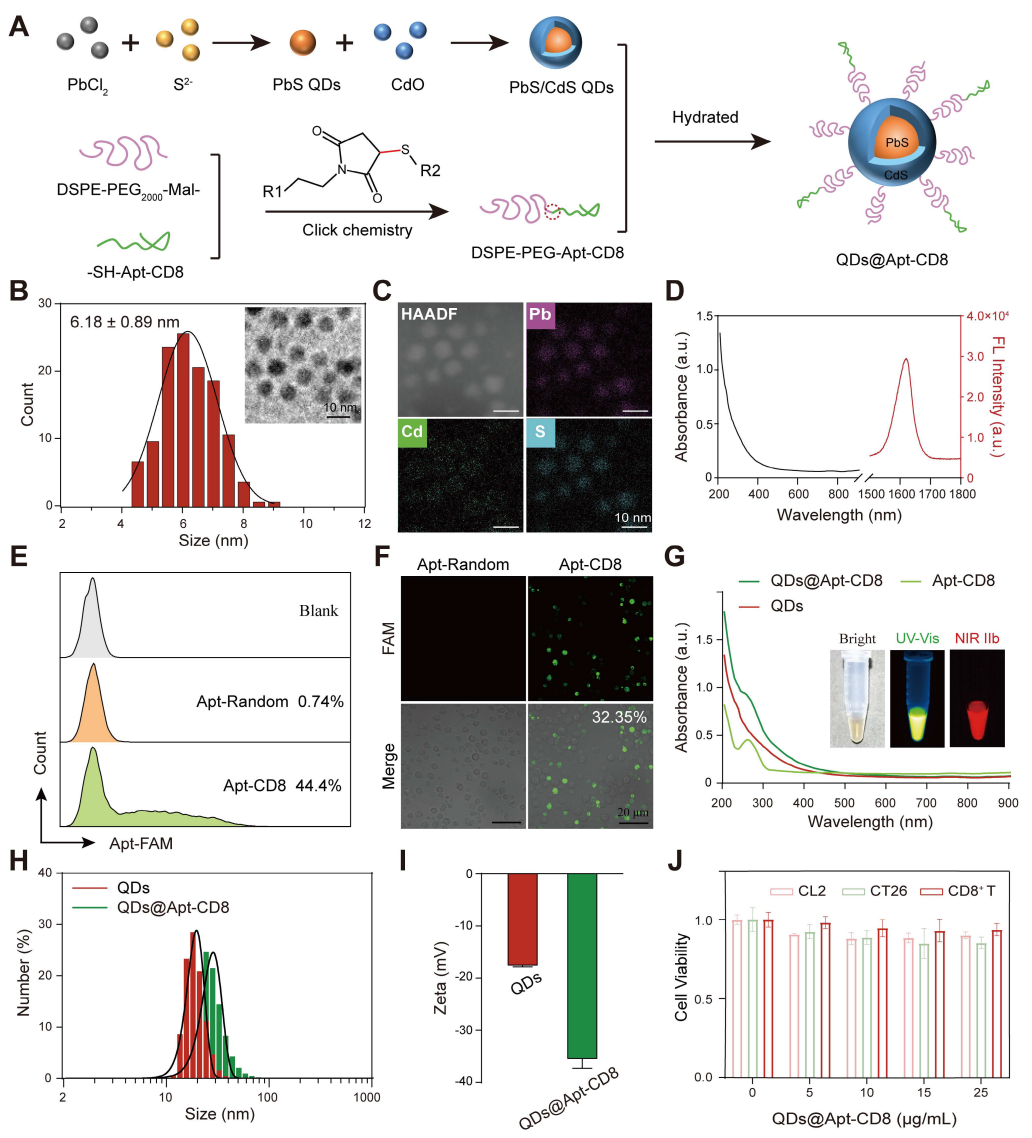
Synthesis and characterization of QDs@Apt-CD8 nanoprobes. (A) Schematic representation of the synthesis process of QDs@Apt-CD8. (B) Size distribution of PbS/CdS QDs (n = 120) along with a representative transmission electron microscopy (TEM) image (inset). (C) High-angle annular dark field TEM (HAADF) image and energy dispersive spectroscopy (EDS) elemental mapping of PbS/CdS QDs. (D) Absorption (black curve) and emission (red curve) spectra of PbS/CdS QDs. (E) Flow cytometry (FCM) profile of CD8^+^ T cells incubated with Apt-CD8 or Apt-Random for 30 min (gated by FAM labeled aptamers). (F) Confocal images of Apt-CD8 and Apt-Random incubated for 30 min with T cells. (G) UV-vis absorption spectra of QDs, Apt-CD8, QDs@Apt-CD8; the inset shows bright field, visible and NIR-IIb fluorescence images of QDs@Apt-CD8. (H) Hydrodynamic size distribution and (I) zeta potential of QDs and QDs@Apt-CD8. (J) Cell viability of CL2, CT26 and CD8^+^ T cells treated with various doses of QDs@Apt-CD8 after 24 h incubation (n = 3).

The QDs@Apt-CD8 nanoprobe was constructed with a CD8 aptamer (Apt-CD8) modified polyethylene glycol (PEG-Apt-CD8) coated onto PbS/CdS QDs by a self-assembly method (Figure 1A). To prepare the QDs@Apt-CD8, PbS/CdS QDs were first synthesized as previously described 37. Transmission electron microscopy (TEM) images confirmed that the as-prepared PbS/CdS QDs exhibited a well-dispersed quasi-spherical morphology with an average diameter of approximately 6.18 ± 0.89 nm (Figure 1B). Meanwhile, energy dispersive spectroscopy (EDS) element mappings revealed that the QDs were composed of Pb, Cd and S (Figure 1C), further validating the successful PbS/CdS core-shell structure. As expected, the PbS/CdS QDs demonstrated a strong fluorescent signal in the NIR-IIb window (1500-1700 nm), showcasing an excellent NIR-IIb fluorescence emission property (Figure 1D).

Although the NIR-IIb luminescence of PbS/CdS QDs is ideally suited for *in vivo* imaging, molecular imaging relies on the targeting capability, stability, and biocompatibility of the QDs in aqueous and biological environments without aggregation or toxicity. The PEGylated outer layer enhances the hydrophilicity and water solubility of the QDs while providing active groups to permit the conjugation of biological ligands for molecular imaging. DSPE-PEG is a non-toxic and biodegradable amphiphilic polymer, which has been approved by the FDA for clinic. Furthermore, the CD8 aptamer has been developed to isolate and purify CD8^+^ T cells due to its high affinity for the T-cell marker CD8 36. Thus, we employed the CD8 aptamer (Apt-CD8) as a recognition ligand for *in vivo* molecular imaging.

Here, as illustrated in Figure 1A, sulfhydryl-modified Apt-CD8 (-SH-Apt-CD8) was conjugated to maleimide-modified DSPE-PEG (DSPE-PEG-Mal-) by a click chemical reaction, forming an amphiphilic polymer with a targeting group (PEG-Apt-CD8). Finally, the hydrophilic QDs@Apt-CD8 were prepared by coating the surface of the PbS/CdS QDs with PEG-Apt-CD8 through the hydrophilic and hydrophobic interaction.

To verify the feasibility of Apt-CD8 for targeting CD8^+^ T cells, we selected FAM-labeled Apt-CD8, FAM-labeled Apt-random (as a negative control) and PE-labeled anti-CD8 (as a positive control) to incubate with CD8^+^ T cells for 30 min. As shown in Figure 1E, the positive rate of Apt-CD8 (44.4%) was significantly higher than that of Apt-random (0.74%), corroborated by the positive control results (anti-CD8, 39.8%, Figure S1). In addition, fluorescence images further demonstrated that the fluorescence signals of Apt-CD8 were significantly greater than those of Apt-random. The positive rate of Apt-CD8 (32.35%), representing the proportion of CD8^+^ T cells binding with Apt-CD8, was largely consistent with the aforementioned results (Figure 1F). Indeed, fluorescence imaging and flow cytometry analyses further confirmed that Apt-CD8 could bind specifically to CD8^+^ T cells, but not to mouse colon carcinoma (CT26) or mouse normal liver (CL2) cells (Figure S2-3). Collectively, these findings indicate the high selectivity and specificity of Apt-CD8 for accurately recognizing CD8^+^ T cells.

Next, the ultraviolet-visible spectra of QDs@Apt-CD8 exhibited a characteristic absorption peak at 260 nm, attributed to the successful coating of Apt-CD8 (Figure 1G). Meanwhile, the fluorescence images captured the representative signals of PbS/CdS QDs (NIR-II) and Apt-CD8 (FAM-labeled, UV-Vis), confirming the successful synthesis of QDs@Apt-CD8. Additionally, QDs@Apt-CD8 demonstrated an excellent photo-stability under 808 nm laser irritation (Figure S4), facilitating its application in NIR-II fluorescence imaging 38. Furthermore, dynamic light scattering (DLS) revealed a higher hydrated size of QDs@Apt-CD8 (38.22 ± 12.54 nm) in aqueous solution compared to that of the QDs (24.32 ± 6.69 nm, Figure 1H), and a uniformed size and morphology were confirmed by TEM (Figure S5). Moreover, the hydrodynamic size of QDs@Apt-CD8 remained largely unchanged in physiological environments (PBS and 10% serum in PBS) over 108 h, indicating an excellent colloidal stability, which is beneficial for NIR-II i*n vivo* imaging (Figure S6). Due to the surface coating of Apt-CD8 (which imparts negative charges), the zeta potential of QDs@Apt-CD8 significantly changed from -17.8 ± 0.44 mV (QDs) to -35.7 ± 1.99 mV (Figure 1I), which is considered a reasonable value for the blood stability. Besides, the cytotoxicity of QDs@Apt-CD8 was assessed through an *in vitro* cell proliferation assay. As depicted in Figure 1J, the QDs@Apt-CD8 exhibited minimal toxicity to CL2 cells, CT26 cells and CD8^+^ T cells at Pb^2+^ concentrations of up to 25 μg/mL. Therefore, a dose of 25 μg/mL was used for further evaluations *in vitro*.

### NIR-II fluorescence imaging of CD8^+^ T cells *in vitro*

**Figure 2:**
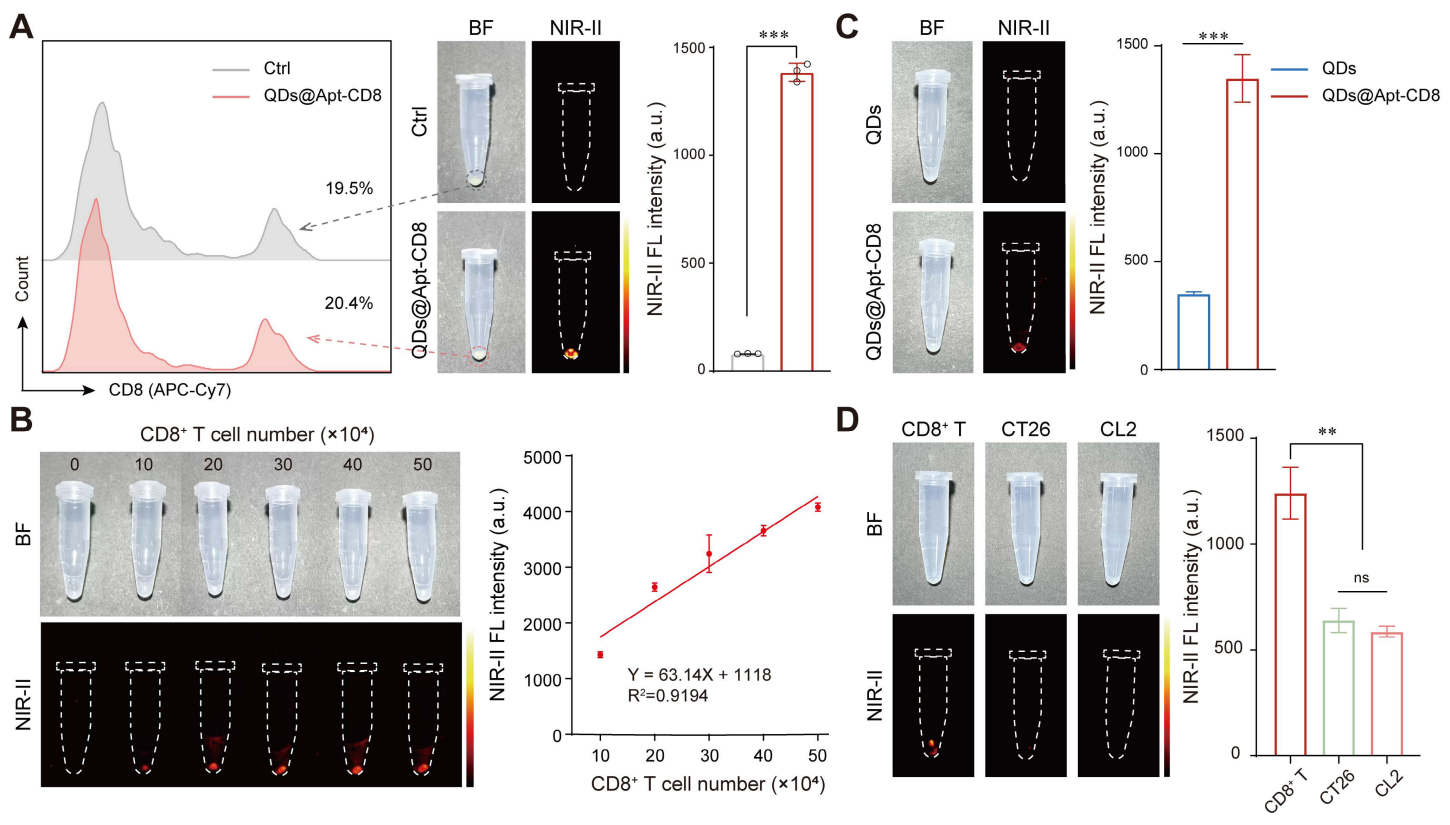
NIR-II fluorescence imaging of CD8^+^ T cells *in vitro*. (A) FCM profiles of CD8^+^ T cells (gated by the APC-Cy7 labeled anti-CD8), alongside *in vitro* NIR-II fluorescence images and the mean fluorescence intensity of CD8^+^ T cells after 30 min incubation with QDs@Apt-CD8 or PBS (as a negative control). (B) *In vitro* NIR-II fluorescence images of CD8^+^ T cells after incubation with QDs@Apt-CD8 for 30 min, with corresponding semi-quantitative analysis (n = 3). (C) *In vitro* NIR-II fluorescence images and mean fluorescence intensity of CD8^+^ T cells after 30 min incubation with QDs and QDs@Apt-CD8 (n = 3). (D) *In vitro* NIR-II fluorescence images and mean fluorescence intensity of CD8^+^ T, CT26 and CL2 cells after 30 min incubation with QDs@Apt-CD8 (n = 3).

To further verify the CD8^+^ T cell-targeting capability of QDs@Apt-CD8, CD8^+^ T cells were isolated from mouse spleen and incubated with QDs@Apt-CD8. The Apt-CD8 on the surface of QDs@Apt-CD8 facilitates high-affinity interactions with CD8 molecular (a representative biomarker for CD8^+^ T cell), endowing it the ability to target these cells selectively. The NIR-II fluorescence images clearly demonstrated that QDs@Apt-CD8 preferentially bound to CD8^+^ T cells, as indicated by the strong fluorescence signals emitted from the cells (Figure 2A). The fluorescence intensity gradually increased with the number of CD8^+^ T cells, exhibiting a good linear relationship in the range of 10 ~ 50 × 10^4^ cells (Figure 2B and Figure S7). In contrast, cells treated with non-functionalized QDs exhibited very weak fluorescence under the same conditions (Figure 2C). By comparing the binding affinity of QDs@Apt-CD8 and QDs to CD8^+^ T cells, it indicated that the targeting ability of QDs@Apt-CD8 is attributed to the surface ligand (Apt-CD8). Moreover, the QDs@Apt-CD8 was incubated with CD8^+^ T cells, CT26 cells and mouse breast cancer (4T1) cells. As expected, the NIR-II fluorescence intensity of CD8^+^ T cells was significantly higher than that of CT26 or 4T1 cells (Figure 2D), confirming the high selectivity and specificity of QDs@Apt-CD8.

Thus, the QDs@Apt-CD8 shows great potential for *in vivo* imaging of CD8^+^ T cells, due to its excellent NIR-IIb luminescence, high selectivity and specificity, stability and biocompatibility in physiological environments.

### *In vivo* distribution and pharmacokinetics of QDs@Apt-CD8

**Figure 3:**
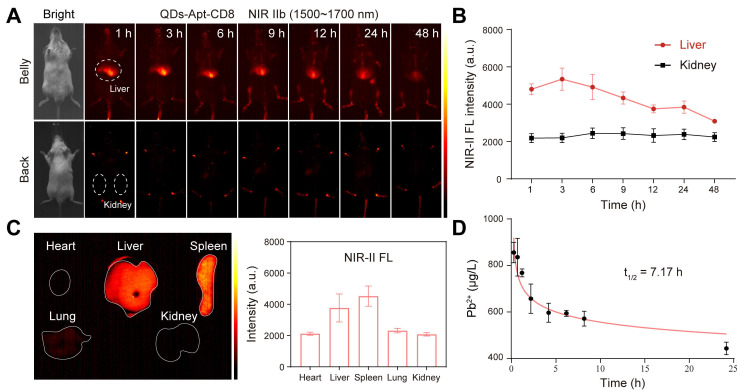
Tissue distribution and metabolism of QDs@Apt-CD8 in healthy BALB/c mice. (A) *In vivo* NIR-II fluorescence imaging of the healthy BALB/c mice. (B) Mean fluorescence intensity in the liver and kidneys after tail vein injection of QDs@Apt-CD8 at various time points (1 h, 3 h, 6 h, 9 h, 12 h, 24 h and 48 h) (n = 3). (C) *Ex vivo* fluorescence images and mean fluorescence intensity of major organs after tail-vein injection of QDs@Apt-CD8 for 48 h. (D) Concentration of Pb^2+^ in blood at 5 min, 30 min, 1 h, 2 h, 4 h, 6 h, 8 h and 24 h following tail intravenous injection of QDs@Apt-CD8 (n = 3).

NIR-IIb fluorescence imaging (at 1500~1700 nm) allows for deep-tissue penetration 39, enabling dynamic and noninvasive *in vivo* imaging of the whole body, major organs, and even blood vessels. This capability facilitates the investigation of the pharmacokinetics, bio-distribution and excretion of QDs@Apt-CD8. The vessels of BALB/c mice were clearly visible within ~5 min post-injection of QDs@Apt-CD8 via tail-vein (Figure S8). The NIR-IIb fluorescence signals in liver rapidly increased within 3 h, and gradually decreased over time, while no distinct fluorescence signals were detected in kidney within 48 h post-injection (Figure 3A, B), indicating a biliary excretion pathway of QDs@Apt-CD8. Notably, significant luminescence signals were still observed in liver and spleen of the dissected mice 48 h after the tail vein injection (Figure 3C), suggesting that QDs@Apt-CD8 primarily accumulated in liver and spleen with minimal retention in other organs such as the heart, lungs and kidneys. The half-life of QDs@Apt-CD8 in blood was 7.17 h, indicating accumulation from blood circulation into these organs (Figure 3D). Additionally, we found that the signal intensity in spleen was higher than that in liver (Figure S9), likely due to the spleen, as an important immune organ, containing a large number of CD8^+^ T cells.

### *In vivo* NIR-II fluorescence imaging of tumor infiltrating CD8^+^ T cells

**Figure 4:**
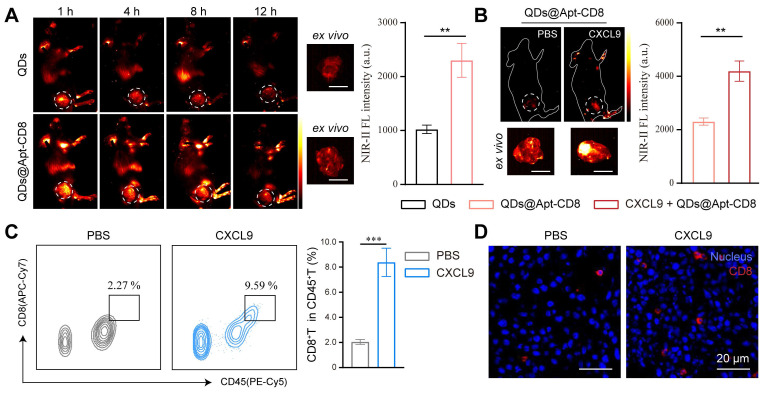
*In vivo* NIR-II fluorescence imaging of tumor infiltrating CD8^+^ T cells. (A) *In vivo* NIR-II fluorescence images of CT26 tumor-bearing mice recorded at different time points after tail vein injection of QDs@Apt-CD8 and QDs, along with *ex vivo* NIR-II fluorescence images of tumor and corresponding fluorescence intensity at 12 h (scale bar, 5 mm). (B) *In vivo* and *ex vivo* NIR-II fluorescence images of tumors from mice treated with CXCL9 or PBS after intravenous injection of QDs@Apt-CD8 for 12 h, along with associated fluorescence intensity (scale bar, 5 mm). (C) Flow cytometry profiles and statistical analysis of CD8^+^ T cells in tumors from mice treated with CXCL9 or PBS for 12 h. (D) Typical immunofluorescence images of tumor-infiltrating CD8^+^ T cells (red) after receiving the indicated treatments. (**p < 0.01, ***p < 0.001, n = 3, one-way ANOVA followed by Tukey`s multiple comparison test.)

To evaluate the *in vivo* NIR-II fluorescence imaging ability of QDs@Apt-CD8, a subcutaneous CT26-tumor model, known for its high immunogenicity, was employed to demonstrate the *in vivo* imaging of tumor-infiltrating CD8^+^ T cells. QDs@Apt-CD8 and QDs (as a negative control) were injected into the tail vein of CT26 tumor-bearing BALB/c mice, and time-dependent whole-body NIR-II fluorescence imaging was performed using a 1500 nm long-pass filter and 1700 nm short-pass filter to reduce tissue auto-fluorescence and improve the signal-noise ratio. As shown in Figure 4A, QDs@Apt-CD8-injected mice exhibited a sustained NIR-II fluorescence signal in the tumor region that remained detectable for approximately 8 h post-injection. The fluorescence signal significantly weakened at 12 h and the tumor contour can be distinctly distinguished from surrounding normal tissues, likely due to the active transport and retention (ATR) effect of nanoparticles (NPs) with the Apt-CD8 as an active targeting ligand 40, 41. Moreover, the *ex vivo* NIR-II fluorescence signal intensity in tumors from the QDs@Apt-CD8-treated group at 12 h post-injection was significantly higher than that of the free QDs-treated group, in which the QDs were non-targeted, lacking Apt-CD8 conjugation but coated with phospholipids. The fluorescence signals from the free QDs exhibited a gradual decline, with much weaker signals in the tumor region compared to QDs@Apt-CD8, indicating that the non-targeted free QDs were readily metabolized and eliminated from the body due to their restricted tumor retention.

To further demonstrate the NIR-II fluorescence imaging capability of QDs@Apt-CD8 for tumor infiltrating CD8^+^ T cells, we intratumorally injected chemokine CXCL9 (10 µg/mL, 2 times) into the tumor of CT26-bearing BALB/c mice 48 h prior to the QDs@Apt-CD8 injection. CXCL9 is one of the ligands of the chemokine receptor CXCR3, and its binding with CXCR3 can recruit CD8^+^ T cells to infiltrate the tumor site 42, 43. As expected, the signal intensity of QDs@Apt-CD8 in tumors from CXCL9-treated mice was 1.85 times higher than that in the control group injected with the same amount of PBS (Figure 4B), attributed to the accumulation of CD8^+^ T cells in tumor via the chemotaxis effect of CXCL9. Furthermore, flow cytometry revealed that CXCL9 significantly increased the infiltration rate of CD8^+^ T cells in tumor from 2.27% to 9.59% (Figure 4C and Figure S10), which was highly consistent with the NIR-II fluorescence imaging results. Additionally, conventional *ex vivo* immunofluorescence (IF) experiments showed a significant increase in the number of tumor-infiltrating CD8^+^ T cells (red fluorescence) in the CXCL9-treated group (Figure 4D and Figure S11), confirming that CXCL9 promotes the tumor infiltration of CD8^+^ T cells. These results suggested that QDs@Apt-CD8 hold the potential to serve as an *in vivo* imaging probe for tumor-infiltrating CD8^+^ T cells, highlighting its significant promise for clinically specific diagnosis.

### *In vivo* NIR-II two-plex molecular imaging for PD1 markers and CD8^+^ T cells

Although tumor-infiltrating CD8^+^ T cells play important roles in tumor immunotherapy, PD1 is a critical biomarker that reflects the immune status of CD8^+^ T cells, influencing the efficacy of immunotherapy 44. Dynamic monitoring of the bio-distribution of CD8^+^ T cells and PD1 expression in tumors, can shed light on the activation and migration patterns of CD8^+^ T cells in response to immune checkpoint blockade therapy. Thus, we further developed a NIR-II two-plex fluorescence imaging approach to non-invasively and simultaneously map CD8^+^ T cells and PD1 *in vivo*. The limited photon scattering and diminished autofluorescence in the NIR-II window ranging (1000-1400 nm and 1500-1900 nm) provide superior penetration depth and lower background signals for *in vivo* imaging 45.

We innovatively selected a dual NIR-II probe pair: ICG, an FDA approved near-infrared dye, and the PbS/CdS QDs for NIR-IIa (1000-1200 nm) and NIR-IIb (1500-1700 nm) imaging, respectively. Apt-PD1 was designed to specifically recognize the PD1 molecule 46, and flow cytometry demonstrated that the positive rate of Apt-PD1 was significantly higher than that of Apt-random (Figure S12). Furthermore, Apt-PD1 only bound to CD8^+^ T cells and not to CT26 or CL2 cells (Figure S13), confirming its specificity for the PD1 molecule on the surface of T cells. Additionally, ICG-Apt-PD1 exhibited minimal toxicity to 4T1 cells, CT26 cells and CD8^+^ T cells at concentrations up to 1.0 μM (Figure S14), indicating excellent biosafety. The NIR-II FL intensity of ICG-Apt-PD1 under continuous 808 nm laser irradiation remained stable over 5 min (Figure S15), demonstrating good photostability for both *in vitro* and *in vivo* NIR-II fluorescence imaging. NIR-II imaging further confirmed the ability of ICG-Apt-PD1 to target PD1 (Figure S16). Pharmacokinetics *in vivo* demonstrated that the half-life of ICG-Apt-PD1 in blood was approximately 20 min (Figure S17), and *ex vivo* NIR-II fluorescence imaging revealed that ICG-Apt-PD1 primarily accumulated in the kidneys with minimal retention in other organs (Figure S18), suggesting a possible renal metabolic pathway.

**Figure 5:**
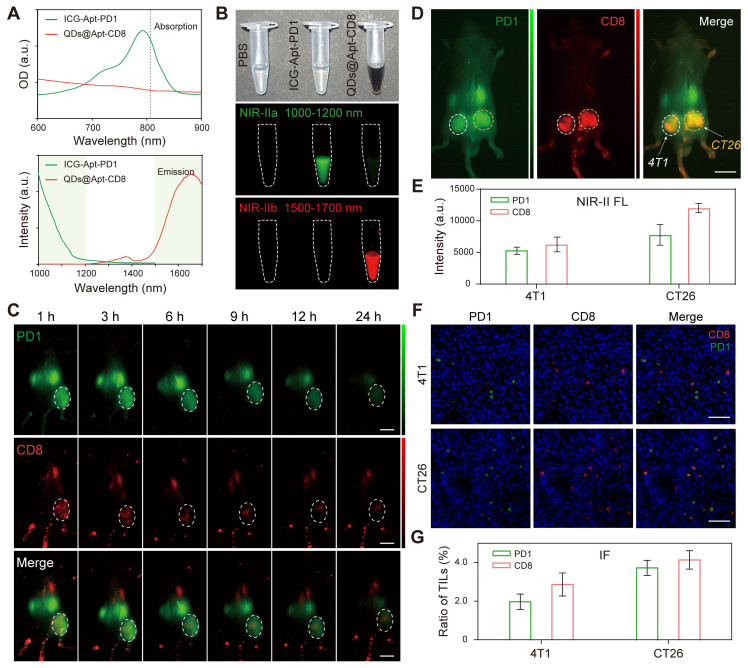
*In vivo* NIR-II two-plex molecular imaging for PD1 markers and CD8^+^ T cells. (A) Absorption (above) and emission (below) spectra of QDs@Apt-CD8 (red curve) and ICG-Apt-PD1 (green curve). The dashed vertical line indicates the excitation wavelength (at 808 nm), and the shaded areas represent the detection ranges for NIR-IIa (1000-1200 nm) and NIR-IIb (1500-1700 nm) in the two-plex NIR-II fluorescence imaging. (B) Color photograph and NIR-II imaging of QDs@Apt-CD8 and ICG-Apt-PD1 in PBS buffer in the NIR-IIa (1000-1200 nm) and NIR-IIb (1500-1700 nm) windows, respectively. (C) *In vivo* NIR-II two-plex fluorescence imaging of CT26 tumor-bearing mice (scale bar, 5 mm). The mice were intravenously injected with QDs@Apt-CD8 and ICG-Apt-PD1, and were imaged at various time points with 808 nm excitation. (D) *In vivo* NIR-II two-plex fluorescence imaging and (E) the corresponding fluorescence intensity of tumor regions from bilateral tumor-bearing mice after the intravenous injection of QDs@Apt-CD8 and ICG-Apt-PD1 for 12 h (left, 4T1 tumor model; right, CT26 tumor model; scale bar, 5 mm). (F) *Ex vivo* immunofluorescence images (scale bar, 40 μm) and (G) statistical analysis of tumor regions from bilateral tumor-bearing mice.

As illustrated in Figure 5A, the NIR-II two-plex imaging probes could be excited by an 808 nm laser and exhibited non-overlapping emission spectra. The fluorescence signals were distinctly captured in the wide-field NIR-II images using 808 nm laser excitation combined with long-pass (LP) and short-pass (SP) emission filters (Figure 5B). Specifically, the NIR-IIa fluorescence signals from ICG-Apt-PD1 were recorded by a NIR-II imaging system equipped with an InGaAs CCD camera and 1000 nm LP plus 1200 nm SP emission filters, allowing for a distinct 1000-1200 nm detection channel without any QDs fluorescence. Similarly, for the NIR-IIb fluorescence imaging, we employed 1500 nm LP plus 1700 nm SP emission filters to capture the fluorescence signals (1500-1700 nm) of QDs@Apt-CD8.

Next, we further explored the two-plex NIR-II fluorescence imaging capability for PD1 markers and CD8^+^ T cells *in vivo*. A mixture of ICG-Apt-PD1 and QDs@Apt-CD8 was injected into CT26 tumor-bearing BALB/c mice via the tail vein. We first performed *in vivo* wide-field NIR-II imaging of the entire body to assess the accumulation of ICG-Apt-PD1 and QDs@Apt-CD8 in tumor at various time points post-injection (p.i.) in both NIR-IIa and NIR-IIb channels. Thanks to their outstanding biocompatibility and targeting capabilities, strong signals in the CT26 tumor were observed in the NIR-IIa channel for PD1 (green) and in the NIR-IIb channel for CD8^+^ T cells (red) at approximately 3 h p.i. (Figure 5C). The emissions from ICG and QDs in the tumor region gradually decreased over time. Similar results were confirmed in the H22 (mouse hepatoma-22) tumor model, further demonstrating the method's capability to evaluate CD8^+^ T cells and PD1 levels *in vivo* (Figure S19).

Upon zooming in for high-magnification imaging of the tumor, PD1 molecules were clearly visualized in the NIR-IIa channel (ICG-Apt-PD1), displaying a relatively uniform signal distribution in tumor (Figure S20). In contrast, the NIR-IIb channel (QDs@Apt-CD8) exhibited stronger signals primarily at the tumor periphery, extending inward (Figure S21). This finding is consistent with *ex vivo* analysis via immunofluorescence (Figure S22), indicating that CD8^+^ T cells predominantly infiltrated the periphery of the CT26 tumor, potentially hindered by the immunosuppressive microenvironment inside solid tumors.

Although not fully addressed the more precise bio-distribution of tumor infiltrating lymphocytes (TILs) in the current study, our NIR-II two-plex fluorescence imaging method enables *in vivo* imaging of independent channels, allowing for the simultaneous mapping of complex biological events. For example, we performed tumor immunophenotyping on solid tumors derived from different cancer types by *in vivo* NIR-II two-plex molecular imaging. Targeted and non-targeted versions of the probes were employed to evaluate CD8^+^ T cells and PD1 levels in the BALB/c mice bearing 4T1/CT26 subcutaneous bilateral tumors (left, 4T1 tumor model; right, CT26 tumor model). The NIR-II signals in the tumor region for the targeted probes (QDs@Apt-CD8 or ICG-Apt-PD1) decreased slowly over time, while the signals from the non-targeted probes (QDs or ICG-Apt-Random) diminished rapidly (Figure S23). This significant difference underscores the targeting capability of our probes. Moreover, the two-plex NIR-II fluorescence imaging, implemented with a wide-field NIR-II imaging setup, allowed us to observe the biodistribution of CD8^+^ T cells (indicated by QDs@Apt-CD8, red) and PD1^+^ T cells (indicated by ICG-Apt-PD1, green) in the tumor at 12 h post injection (Figure 5D). The fluorescence intensity in CT26 tumor was significantly higher than that in 4T1 tumor for both NIR-IIa (ICG-Apt-PD1) and NIR-IIb (QDs@Apt-CD8) channels, indicating a greater presence of CD8^+^ T cells and PD1 expression in the CT26 tumor compared to the 4T1 tumor (Figure 5E and Figure S24). This suggests that CT26 tumors exhibit a more active immunophenotype, characteristic of "hot" tumors, while 4T1 tumors display fewer infiltrating lymphocytes, aligning with the immunophenotype of "cold" tumors. Flow cytometry further confirmed these findings, revealing that the proportion of CD8^+^ PD1^+^ T cells in the CT26 tumor (20.7%) was significantly higher than that in the 4T1 tumor (12.9%), consistent with the *in vivo* NIR-II fluorescence imaging results (Figure S25).

Finally, we compared our two-plex *in vivo* imaging with the traditional immunofluorescence (IF) method, commonly used for *ex vivo* immunophenotyping. IF was conducted on the tumor tissues from the same batches as those used for *in vivo* imaging, and images were analyzed using ImageJ software to quantify the biomarker expression levels (Figure. 5D, F and Figure S26). Statistically, the two methods demonstrated a degree of correlation in biomarker expression patterns of tumor immunophenotypes (Figure. 5E and G). However, significant heterogeneity has been documented among IF images staining the same receptor across multiple tissue samples collected from the same mice 47. In contrast, *in vivo* NIR-II two-plex molecular imaging allows for the non-invasive quantification of the entire tumor, reducing uncertainties associated with biopsy, sample processing, and scoring procedures, while also eliminating the risk of tumor cell reseeding following biopsy.

### Two-plex NIR-II *in vivo* imaging of anti-PD1 immunotherapy

**Figure 6:**
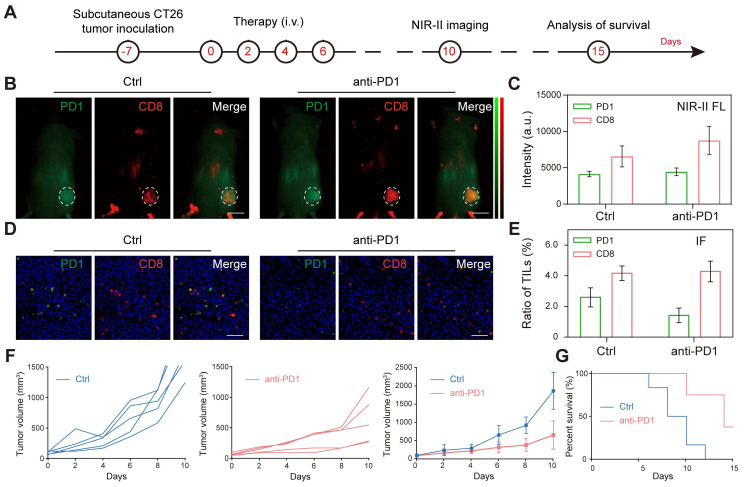
Two-plex NIR-II *in vivo* imaging of anti-PD1 immunotherapy. (A) Schematic illustration of the tumor immunotherapy procedure and NIR-II fluorescence imaging of the subcutaneous CT26 tumor model. (B, C) *In vivo* NIR-II two-plex fluorescence imaging and corresponding fluorescence intensity (scale bar, 5 mm), (D, E) *ex vivo* immunohistochemistry assay of tumor regions from CT26 tumor-bearing mouse (scale bar, 40 μm). (F) Tumor volume and (G) survival curve of tumor-bearing BALB/c mice after receiving different treatments as indicated.

The efficacy of tumor immunotherapy is closely linked to the expression levels of CD8 and PD1 in the tumor microenvironment 6, 48. To further investigate this relationship, we conducted two-plex NIR-II *in vivo* molecular imaging of CD8 and PD1 in tumor in response to anti-PD1 immunotherapy. CT26-bearing mice were randomly divided into two groups (n = 5) and received tail-vein injections of either PBS or anti-PD1 (3.0 mg/kg) for a total of 4 times, every 2 days. Four days later, we administered QDs@Apt-CD8 and ICG-Apt-PD1 intravenously, and performed wide-field two-plex NIR-II *in vivo* imaging at 12 h post injection (Figure. 6A). *In vivo* imaging revealed that the intensity of CD8 signals in the tumor region was significantly higher in the anti-PD1-treated mice (anti-PD1) compared to the control group (Ctrl), while there was not much difference in PD1 levels between the two groups (Figure. 6B and C). Flow cytometric analysis corroborated these findings, showing increased expression levels of CD8 and PD1 in the anti-PD1 group (anti-PD1) compared to the control group (Ctrl), aligning closely with the results from two-plex NIR-II *in vivo* imaging (Figure S27). Furthermore, we performed *ex vivo* immunofluorescence (IF) using the tumor tissues from the same batches as those used for *in vivo* imaging, as displayed in Figure 6D and Figure S28, the expression level of PD1 in the anti-PD1 treated group (anti-PD1) was significantly lower than that in the non-treated group (Ctrl), while there was no notable difference in the number of tumor-infiltrating CD8^+^ T cells (Figure 6E). Due to tumor heterogeneity and the inherent features of the imaging techniques (*in vivo* imaging assesses the overall status of the tumor, while immunofluorescence emphasizes local molecular features), some discrepancies arose in evaluating CD8 and PD1 expression in the tumor microenvironment. Nevertheless, the trends in relative expression levels of CD8 and PD1 after anti-PD1 treatment were highly consistent. Notably, we observed a significant decrease in the PD1/CD8 ratio (Figure S29), indicating that after anti-PD1 immunotherapy, the expression level of PD1 in the tumor microenvironment decreases while the immune cytotoxicity of CD8^+^ T cells increases 44. As expected, tumor progression was distinctly suppressed after anti-PD1administration (Figure 6F), and anti-PD1 treatment significantly extended the survival of tumor-bearing mice compared to the control group (Figure 6G). Therefore, two-plex NIR-II *in vivo* imaging can serve as a powerful tool for monitoring the expression level of CD8 and PD1 in the tumor microenvironment and predicting the efficacy of tumor immunotherapy.

### *In vivo* biocompatibility evaluation

**Figure 7:**
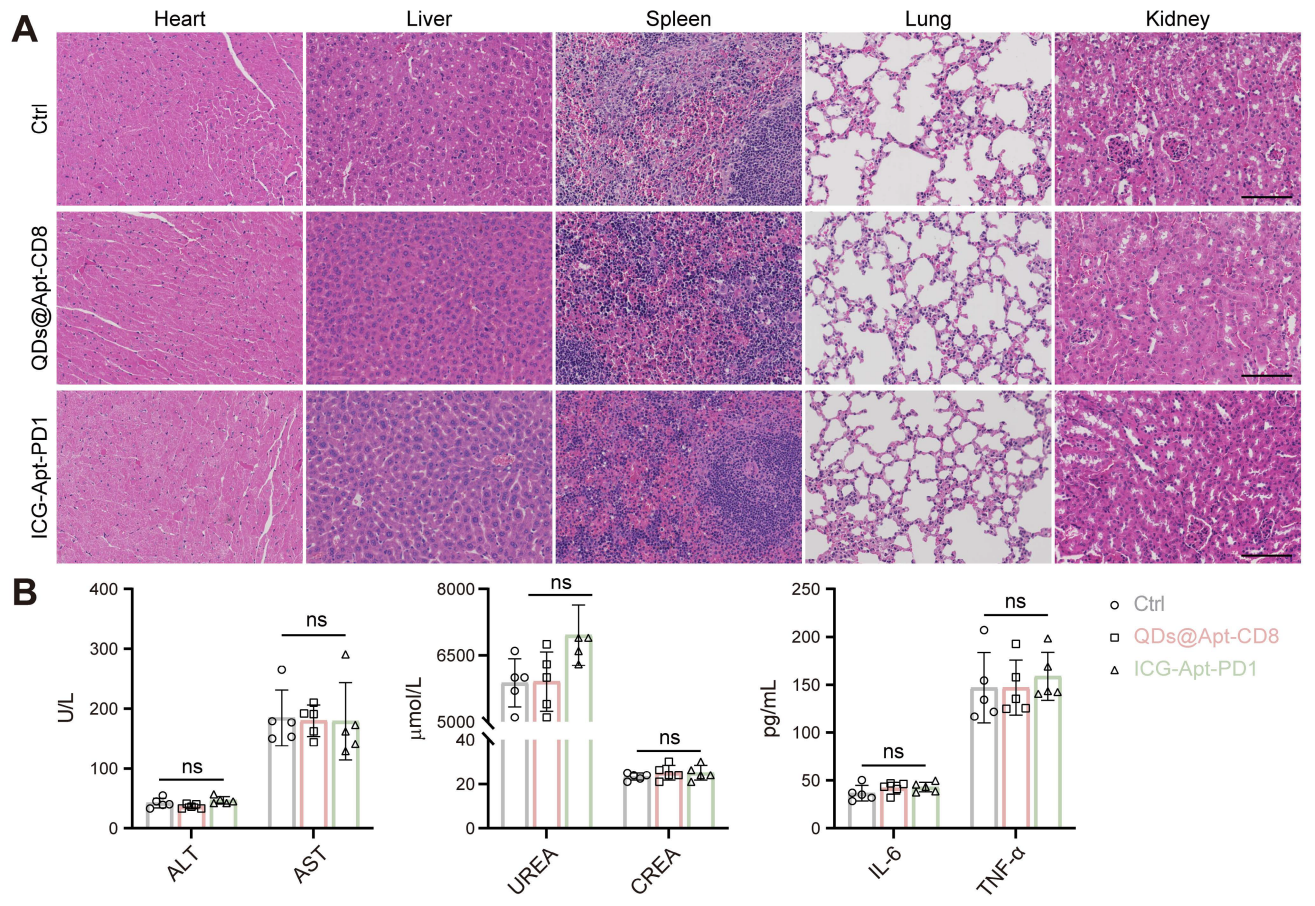
Biosafety assessment of the QDs@Apt-CD8 and ICG-Apt-PD1 probes. (A) Histopathological assays of major organs from the mice receiving QDs@Apt-CD8, ICG-Apt-PD1 or PBS for 14 days (scale bar, 200 μm). (B) Blood biochemical and cytokines test of mice after indicated treatments. Data are presented as mean ± S.D. (n = 5, biologically independent replicates, ns represents not significant). Abbreviations: AST, aspartate aminotransferase; ALT, alanine aminotransferase; CREA, creatinine; UREA, urea.

The excellent biocompatibility of NIR-II fluorescent probes is essential for their clinical application. To assess the *in vivo* toxicity of QDs@Apt-CD8 and ICG-Apt-PD1, we collected the serum and major organs (heart, liver, spleen, lung and kidney) from the mice receiving different treatments. Hematoxylin and eosin (H&E) staining of these organs revealed intact cell morphology, with clear nuclei and cytoplasm, and no pathological changes (Figure 7A). Moreover, the serum biochemical indicators (ALT and AST for liver function, UREA and CREA for renal function), and inflammatory factors (TNF-α and IL-6) showed no significant differences across the groups (Figure 7B and Figure S30). The results indicated that QDs@Apt-CD8 and ICG-Apt-PD1 possess excellent biocompatibility with negligible side effects, highlighting their potential for further clinical translation in the field of multiplex NIR-II *in vivo* imaging.

## Conclusion

In summary, we developed *in vivo* two-plex NIR-II molecular imaging of CD8^+^ T cells and PD1 markers to evaluate the tumor immunophenotypes and predict the therapeutic efficacy of immunotherapy. Thanks to its excellent NIR-IIb luminescence, high selectivity and specificity, stability and biocompatibility in physiological environments, QDs@Apt-CD8 demonstrates outstanding capabilities for *in vivo* imaging of tumor infiltrating CD8^+^ T cells. The non-overlapping fluorescence emission of ICG in the NIR-IIa window (1000-1200 nm) and PbS/CdS QDs in the NIR-IIb window (1500-1700 nm) enabled our two-plex *in vivo* molecular imaging technique to dynamically monitor the levels of PD1 and the number of CD8^+^ T cells in tumor. We observed the heterogeneous bio-distributions of PD1 and CD8^+^ T cells across different tumor types, revealing the distinct tumor immunophenotypes. Notably, two-plex *in vivo* molecular imaging suggested that the lower PD1 levels coupled with higher CD8^+^ T cells levels in tumors predicted better anti-tumor effects. Furthermore, the excellent *in vivo* bio-safety with negligible side effects further indicated the potential of QDs@Apt-CD8 and ICG-Apt-PD1 for clinical application. Such *in vivo* noninvasive NIR-II molecular imaging of CD8^+^ T cells and PD1 markers in tumor could complement *ex vivo* biopsy-based diagnostic techniques, such as immunofluorescence (IF) or immunohistochemistry (IHC). In the future, it is possible to develop an *in vivo* tumor immune-scoring algorithm based on the number of tumor-infiltrating CD8^+^ T cells and immune status to afford a more precise prediction for immunotherapeutic response.

## Materials and Methods

**Materials**. Lead chloride (PbCl_2_), chromium oxide (CdO) and sulfur powder were purchased from Sigma-Aldrich. Oleylamine and oleic acid were obtained from Aladdin Reagent (Shanghai) Co., Ltd. DSPE-PEG and DSPE-PEG-Mal (MW ~ 2000) were ordered from Chongqing Yusi medical technology cable Co., Ltd. Cell Counting Kit (CCK-8) were purchased from Dojindo Laboratories (Kumamoto, Japan). The chemokine CXCL9 was obtained from Bio-Techne (Shanghai, China). The CD8^+^ T cell magnetic bead sorting kit was purchased from Miltenyi Biotec. Mouse interleukin-6 (IL-6) ELISA Kit (CSB-E04639m-IS) and tumor necrosis factor (TNF-α) ELISA kit (CSB-E04741m) were obtained from CUSABIO Biotech CO., Ltd. (Wuhan, China). Dulbecco's modified Eagle's medium (DMEM), fetal bovine serum (FBS), KBM 581 medium, RPMI-1640 medium, penicillin and streptomycin were ordered from Thermo Fisher Scientific. Mouse CD8 antibody (anti-CD8, ab217344) and mouse PD1 antibody (anti-PD1, ab214421) were purchased from Abcam (ABCM, USA). All of the antibodies for flow cytometric analysis were obtained from eBioscience (eBioscience, USA). Analytical-grade chemicals were purchased from Sinopharm Chemical Reagent Co. Ltd. The oligonucleotides for aptamers were ordered from Sangon Biotech (Shanghai) Co., Ltd. The sequences of oligonucleotides can be found in Supplementary Table S1.

**Synthesis of QDs@Apt-CD8 nanoprobes**. DSPE-PEG-Apt-CD8 was synthesized via a click chemical reaction between the maleimide group (-Mal) on DSPE-PEG_2000_ and the sulfhydryl group (-SH) on Apt-CD8. Specifically, 1.36 mg of DSPE-PEG-Mal powder (MW ~ 2000) was added into a 1.5 mL EP tube, followed by the addition of 1 mL PBS solution and sonication to ensure complete dissolution. Subsequently, 20 μL Apt-CD8 (100 μM) was introduced, and the mixture was oscillated overnight at room temperature. Afterward, the solution was freeze-dried following dialysis purification.

For further modification, the prepared PbS/CdS QD (5.0 mg) was dissolved in 10 mL of chloroform containing 20 mg of DSPE-PEG and 1 mg of DSPE-PEG-Apt-CD8. The mixture was stirred at room temperature for 30 min, followed by solvent removal under vacuum using a rotary evaporator. The resulting residue was then dissolved in 5 mL of ddH_2_O with sonication. The product was then transferred to a 100 KD ultrafiltration tube, centrifuged at 3500 rpm for 10 min, and washed 2-3 times with ddH_2_O to eliminate the excess polymer chains. The purified product was dissolved in PBS buffer (0.01 M, pH 7.4) and stored at 4 °C.

**Function identification of Apt-CD8 *in vitro*.** For flow cytometry analysis. To verify the selectivity of Apt-CD8, CD8^+^ T cells were incubated with 5% BSA solution for 15 min, and randomly divided into 4 groups (2 × 10^5^ cells in each group): Blank, anti-CD8 antibody (PE), Apt-random-FAM and Apt-CD8-FAM. Following this, a working solution (100 μL) containing either Apt-random-FAM (1 μM), Apt-CD8-FAM (1 μM) or anti-CD8 antibody (PE, 1μL) in 0.5% BSA solution was added to each group and and incubated with CD8^+^ T cells for 30 min. Finally, the CD8^+^ T cells were washed with PBS, re-suspended in 250 μL PBS solution, and measured by flow cytometry (BD, FACSVerse, USA). To assess the specificity of Apt-CD8, it was co-incubated with CD8^+^ T cells, CT26 and CL2 cells, following a similar flow cytometry analysis procedure.

For fluorescence imaging analysis, CD8^+^ T cells were incubated with Apt-random-FAM and Apt-CD8-FAM for 30 min, respectively. Apt-CD8 was co-incubated with CD8^+^ T cells, CT26 and CL2 cells for the same duration. After centrifugation, 1 × Hoechst 33342 dye (1 mL) was added and incubated for 15 min. The cells were washed and re-suspended in PBS buffer. The fluorescence signal (FAM dye) was observed by confocal laser microscopy (CLSM, Zeiss LSM780).

**NIR-II fluorescence imaging of CD8^+^ T cells *in vitro*.** The QDs@Apt-CD8 and QDs working solution were prepared using fresh serum-free RPM1640 medium (a final concentration of 25 μg/mL, measured by Pb^2+^).

To verify the selectivity of QDs@Apt-CD8, CD8^+^ T cells were incubated with QDs@Apt-CD8 and QDs at 4℃ for 30 min. To verify the specificity of QDs@Apt-CD8, QDs@Apt-CD8 was co-incubated with CD8^+^ T cells, CT26 and CL2 cells at 4℃ for 30 min. To evaluate the ability of QDs@Apt-CD8 for semi-quantitative analysis of CD8^+^ T cells *in vitro*, varying numbers of CD8^+^ T cells (0, 2, 4, 6, 8, 10 × 10^4^) were co-incubated with QDs@Apt-CD8 at 4℃ for 30 min. The cells were washed with PBS to remove the excess fluorescent probes. The NIR-II fluorescence images were obtained by an UniNano NIR-II imaging system (imaging conditions: 808 nm excitation, 1,500-1,700 nm detection, exposure time, 200 ms).

***In vivo* distribution of QDs@Apt-CD8.** To explore the tissue distribution of QDs@Apt-CD8, the BALB/c mice (6-8 weeks) were administered with QDs@Apt-CD8 (25 mg/kg) via tail-vein injection. *In vivo* fluorescence signals were recorded at various time points (1 h, 3 h, 6 h, 9 h, 12 h, 24 h and 48 h) using the UniNano NIR-II imaging system (808 nm excitation, 1,500-1,700 nm detection, exposure time, 200 ms). Major organs (heart, liver, spleen, lung and kidney) were collected and imaged following the same procedure.

***In vivo* NIR-II fluorescence imaging of tumor infiltrating CD8^+^ T cells.** The BALB/c mice (6-8 weeks) were subcutaneously injected with CT26 cells (1 × 10^6^). While the tumor volume reached 300-400 mm^3^, the CT26-bearing mice were randomly divided into two groups (n = 3) and injected with either QDs or QDs@Apt-CD8 (20 mg/kg) via the tail-vein. Then, NIR-II fluorescence images were recorded at various time points post injection by an UniNano NIR-II imaging system. Tumors were collected at 12 h after injection and performed *ex vivo* NIR-II fluorescence imaging (808 nm excitation, 1,500-1,700 nm detection, exposure time, 200 ms).

To further verify the capability of QDs@Apt-CD8 for *in vivo* NIR-II fluorescence imaging, CT26-bearing mice were randomly divided into two groups (n = 3), and intratumorally injected with chemokine CXCL9 (10 µg/mL, 50 µL) and equal amount of PBS (Ctrl). Then, *in vivo* and *ex vivo* NIR-II fluorescence imaging were performed using the same procedures as described above.

***In vivo* toxicity evaluation.** The BALB/c mice (6-8 weeks) were injected with PBS, QDs@AptCD8 (20 mg/kg) and ICG-Apt-PD1 (500 pmol) by tail-vein injection (n = 5), respectively. 14 days after injection, the blood was collected from the orbital sinus of mice for serum biochemical indicator and blood routine test. The cytokines (TNF-α and IL-6) were measured by ELISA Kit according to the standard protocols. For hematoxylin-eosin (H&E) pathological staining, the visceral organs (heart, liver, spleen, lungs, and kidneys) were immersed in 4% paraformaldehyde and fixed for 24 h. The tissues were then embedded in paraffin, sectioned, and stained with H&E following standard protocols.

## Supplementary Material

Supplementary materials and methods, figures and tables: the selectivity and specificity of Apt-CD8, characterizations for QDs@Apt-CD8, additional NIR-II imaging data, *ex vivo* immunofluorescence images, flow cytometry profiles, histological examination, mass spectrometry analysis, biocompatibility data and corresponding statistical analysis.

## Abbreviations

PD1: programmed death-1
NIR-II: second near infrared window
ICG: indocyanine green
QDs: quantum dots
ICG-Apt-PD1: PD1 aptamer-labeled ICG
QDs@Apt-CD8: CD8 aptamer-labeled QDs
ICB: immune checkpoint blockades
TME: tumor microenvironment
FCM: flow cytometry
Apt-CD8: CD8 aptamer
Apt-random: random aptamer
Apt-PD1: PD1 aptamer
p.i.: post-injection
IF: immunofluorescence
NIR-II FL: near-infrared-II fluorescence imaging
