# Supplementary material for: Two-plex in vivo molecular imaging in the second near-infrared window for immunotherapeutic response
Source: Theranostics. 2025 Mar 19;15(10):4481–94. doi: 10.7150/thno.108880 (PMC11984393; doi:10.7150/thno.108880)
Supplement: Supplementary file 1 — Supplementary materials and methods, figures and tables: the selectivity and specificity of Apt-CD8, characterizations for QDs@Apt-CD8, additional NIR-II imaging data, ex vivo immunofluorescence images, flow cytometry profiles, histological examination, mass spectrometry analysis, biocompatibility data and corresponding statistical analysis. [file thnov15p4481s1.pdf]

Supporting information

# Two-plex *in vivo* molecular imaging in the second near-infrared window for immunotherapeutic response

Yupeng Sun,#*[a,b] Rui Li,#[a,c] Yike Cai,[a,c] Yan Liu,[a,c] Peiyuan Wang[a,e] Ming Wu,[a,b] Xiaolong Zhang,[a,b] Naishun Liao,[a] Cuilin Zhang,[a,b] Aixian Zheng,[a,b] Haipo Xu,[a] Rui Zeng,[a] Yongyi Zeng,[a,d] and Xiaolong Liu*[a,b,e]

[a] The United Innovation of Mengchao Hepatobiliary Technology Key Laboratory of Fujian Province
Mengchao Hepatobiliary Hospital of Fujian Medical University
Fuzhou 350025, P. R. China
E-mail: xiaoloong.liu@gmail.com, sunyp_jlu@163.com

[b] Mengchao Med-X Center
Fuzhou University
Fuzhou 350116, P. R. China

[c] College of Biological Science and Engineering
Fuzhou University
Fuzhou, 350116, PR China

[d] Liver Disease Center
The First Affiliated Hospital of Fujian Medical University
Fuzhou 350005, P. R. China

[e] CAS Key Laboratory of Design and Assembly of Functional Nanostructures,
Fujian Institute of Research on the Structure of Matter, Chinese Academy of Sciences
Fuzhou 350002, P. R. China

**DOI:** 10.7150/thno.108880

## Materials and Methods

**Synthesis of PbS/CdS QDs**. The PbS/CdS quantum dots (QDs) were synthesized using a modified procedure (*Proc. Natl. Acad. Sci. U.S.A 2018, 115 (26), 6590-6595*). Specifically, sulfur precursor solution was prepared by mixing 0.08 g (5 mmol) of sulfur powder with 7.5 mL of oleylamine in a three-neck flask at 120 °C, followed by degassing under argon for 30 min and cooling to room temperature. Similarly, lead precursor solution was prepared by mixing 0.834 g (3 mmol) of $PbCl_2$ and 7.5 mL of oleylamine in a three-neck flask, degassing for 30 min under argon at 120 °C and then injecting 2.5 mL of oleic acid while stirring at 120 °C for an additional 30 min (during which the reaction solvent would change from milky white to clear and transparent). Subsequently, the as-prepared sulfur precursor solution (2.25 mL) was quickly injected into the Pb precursor solution (3 mmol of Pb) under stirring, and the mixture was maintained at 160 °C under argon for 30 min. The reaction was then quenched by adding 10 mL of cold hexane and 20 mL of ethanol. The products were collected by centrifugation and resuspended in a mixture of 10 mL of hexane/20 mL of oleic acid. After stirring for 15 min, the mixture was centrifuged at 10000 rpm for 10 min to remove the excess unconverted reactants. This precipitation procedure with oleic acid was repeated 2-3 times until the supernatant was colorless. Finally, the PbS QDs were redispersed in octadecene (ODE).

Cd precursor solution was prepared by mixing CdO (1.2 g, 9.2 mmol), oleic acid (8 mL) and ODE (20 mL) in a three-neck flask at 200 °C under argon for 30 min, followed by cooling to 100 °C. 5 mL of the as-prepared PbS QDs suspended in ODE was bubbled with argon for 10 min, then injected into the Cd precursor and maintained for 30 min at 100 °C under argon. Finally, the reaction was quenched with 5 mL of cold hexane, and the PbS/CdS QDs were precipitated, washed with ethanol 2-3 times, and redispersed in chloroform.

**Characterizations.** The quantification of quantum dots was conducted using inductively coupled plasma (ICP) to determine the concentration of $Pb^{2+}$. The morphology and energy-dispersive X-ray spectroscopy (EDS) elemental mapping of PbS/CdS QDs were obtained by transmission electron microscopy (TEM, FEI Company, Hillsboro, OR). The hydrated-particle size and zeta potential of QDs and QDs@Apt-CD8 were measured via dynamic light scatterings (DLS, Malvern Zetasizer). The UV-vis absorption spectra of QDs, Apt-CD8, QDs@Apt-CD8 was recorded using a multifunctional microplate reader (Spectra Max M5, Molecular Devices). The NIR fluorescence spectra QDs and QDs@Apt-CD8 were acquired using an FLS1000 Photoluminescence Spectrometer (Edinburgh, excited at 808 nm and recorded at 1500-1800 nm). The UV-vis fluorescence image of QDs@Apt-CD8 (where Apt-CD8 was labelled with FAM dye and excited at 488 nm) was obtained by the ChemiDoc™ MP imaging system, while the NIR-IIb fluorescence image of QDs@Apt-CD8 was acquired by an UniNano NIR-II imaging system (imaging conditions: 808 nm excitation, 1500-1700 nm detection, exposure time, 200 ms).

**Isolation of $CD8^+$ T cells.** $CD8^+$ T cells were isolated from the spleen of C57BL/6 mice (6-8 weeks) according to a previously reported method (*Advanced Science, 2024, 2400951*). Briefly, the spleen was collected and immersed in PBS buffer. Firstly, the cells were flushed out from the spleen using a syringe filled with PBS buffer. Then, the cell suspension was filtered through a 40 μm cell-strainer to eliminate the large debris. Afterwards, the cells were centrifuged at 800×g for 5 min, and re-suspended in red blood cell lysis buffer for 5 min at 25℃, followed by a second centrifugation for 5 min at 800×g. Finally, the $CD8^+$ T cells were isolated by a $CD8^+$ T Cell Isolation Kit (Miltenyi Biotec) according to the provided protocol.

**Cell culture**. CT-26 cells (mouse colon cells), 4T1 cells (mouse breast cancer cells) and CL2 cells (mouse normal liver cell line, bnl cl.2) were maintained in DMEM medium supplemented with fetal bovine serum (FBS, 10%) and penicillin-streptomycin (100 U $mL^{-1}$). $CD8^+$ T cells were incubated in KBM 581 medium containing IL-2 (10 ng $mL^{-1}$, R&D systems, MX2918061). Anti-CD3ε (5 μg $mL^{-1}$, biolegend, 100331) and anti-CD28 (2 μg $mL^{-1}$, biolegend, 102112) were only added during the initial activation of the $CD8^+$T cells. All the cells were cultured at 37 ℃ in a humidified atmosphere of 5% $CO_2$ and 95% air humidity.

***In vitro* cytotoxicity assay**. To assess the cytotoxicity of QDs@Apt-CD8, CT26 cells, CL2 cells and $CD8^+$ T cells were cultured in a 96-well plate at a density of $2×10^4$ cells per well and incubated for 24 h. Afterward, the fresh medium containing varying concentrations of QDs@Apt-CD8 (calculated by $Pb^{2+}$, 0, 5, 10, 15, 25, and 50 μg $mL^{-1}$) was added, and the cells were incubated for an additional 24 h. Cell viability was measured via a CCK-8 Kit according to the previously reported methods.

**Animals.** BALB/c mice (male, 6-8 weeks, 20-22 g) were purchased from Shanghai Slack Laboratory Animal Co., Ltd. The animals were conducted in accordance with the guidelines outlined in the Guide for the Care and Use of Laboratory Animals. All the experiments were approved by the Animal Ethics Committee of Mengchao Hepatobiliary Hospital of Fujian Medical University (MCHH-AEC-2022-11).

***In vivo* pharmacokinetics of QDs@Apt-CD8.** To evaluate the pharmacokinetic distribution of QDs@Apt-CD8 in healthy mice, the half-life of QDs@Apt-CD8 in blood was calculated based on the concentration of $Pb^{2+}$ in blood at various time points after injection. Specifically, QDs@AptCD8 (50 mg/kg) was administered intravenously (via tail vein) to the healthy BALB/c mice. Blood samples were collected at 5 min, 30 min, 1 h, 2 h, 4 h, 6 h, 8 h and 24 h post injection to assess blood circulation times based on the $Pb^{2+}$ concentration. The tail vein blood (about 100 μL) of the BALB/c mice was collected and centrifuged at 2000 g for 10 min, the upper serum (20 μL) was then treated with aqua regia, and the concentration of $Pb^{2+}$ in blood was determined by ICP. Finally, the half-life of QDs@Apt-CD8 in blood was calculated by a first-order elimination kinetics curve.

The calculation formula is as follows: $lgC_t=-Kt/2.303+lgC_0$, $t_{1/2}=0.693/K$,

where t is the time, $C_t$ is the drug concentration when t, K is the elimination rate constant, reflecting the elimination rate of drugs in blood, and $t_{1/2}$ is the half-life.

***In vivo* NIR-II fluorescence imaging of tumor infiltrating $CD8^+$ T cells.** BALB/c mice (6-8 weeks) were subcutaneously injected with CT26 cells ($1 \times 10^6$). When the tumor size reached 300-400 $mm^3$, the CT26-bearing mice were randomly divided into two groups (n=3), and injected with either QDs or QDs@Apt-CD8 (20 mg/kg) via tail-vein. The NIR-II fluorescence images of the mice were recorded at various time points post injection using an UniNano NIR-II imaging system. 12 h after injection, the tumors were collected for *ex vivo* NIR-II fluorescence imaging. (808 nm excitation, 1,500-1,700 nm detection, exposure time, 200 ms)

To further validate the reliability of QDs@Apt-CD8, the *ex vivo* tumors were collected to prepare a cell suspension according to the previously reported methods. Briefly, the tumors were digested with collagenase type IV (1 mg $mL^{-1}$), hyaluronidase (0.2 mg $mL^{-1}$) and DNase I (0.02 mg $mL^{-1}$) at 37 ℃ for 2 h. The single-cell suspension was obtained by filtering through a cell-strainer, and flow cytometry (FCM) was used to evaluate the presence of $CD8^+$ T cells in the

tumor (staining with anti-CD8-APC-Cy7 and anti-CD45-PE-Cy5). Additionally, for immunofluorescence analysis of $CD8^+$ T cells in tumor, the harvested tumors were fixed in 4% paraformaldehyde for 24 h, followed by paraffin embedding and section, and immunofluorescence staining according to standard protocols.

***In vivo* NIR-II two-plex fluorescence imaging.** The NIR-II fluorescence images were acquired by an UniNano NIR-II imaging system. For ICG-Apt-PD1, a 1000 nm long-pass (LP) filter combined with a 1200 nm short-pass (SP) emission filter was used for the NIR-IIa channel (1000-1200 nm), imaging conditions: 808 nm excitation, 1000-1200 nm channel, exposure times 200 ms, 1× or 10× magnification objective. For QDs@Apt-CD8, a 1500 nm LP filter paired with a 1700 nm SP emission filter was employed for the NIR-IIb channel (1500-1700 nm), imaging conditions: 808 nm excitation, 1500-1700 nm channel, exposure times 200 ms, 1× or 10× magnification objective.

BALB/c mice (6-8 weeks) were subcutaneously injected with CT26 cells ($1 \times 10^6$). When the tumor size reached 300-400 $mm^3$, the CT26-bearing mice was injected via tail-vein with QDs@Apt-CD8 (20 mg/kg) and ICG-Apt-PD1 (500 pmol). The two-plex NIR-II *in vivo* fluorescence images of the mice were recorded at various time points post injection.

Additionally, a bilateral tumor-bearing mice model was established by subcutaneously injecting CT26 cells ($1 \times 10^6$) on the right side and 4T1 cells ($2 \times 10^6$) on the left side of BALB/c mice (6-8 weeks). When the tumor size reached 300-400 $mm^3$, the mice were injected with QDs@Apt-CD8 (20 mg/kg) and ICG-Apt-PD1 (500 pmol) via tail-vein. The two-plex NIR-II *in vivo* fluorescence images fluorescence images were recorded at 12 h post injection. For flow cytometry analysis, the 4T1/CT26 tumors were collected to prepare a cell suspension, which was analyzed by flow cytometry (BD, FACSVerse, USA) following the previously reported methods (staining with anti-CD45-PE-Cy5, anti-CD8-PE and anti-PD1-PE-Cy7). For immunofluorescence analysis, the harvested tumors were fixed in 4% paraformaldehyde for 24 h, followed by paraffin embedding and section, and immunofluorescence staining according to standard protocols.

**Tumor immunotherapy.** To evaluate the anti-tumor effects of anti-PD1 (anti-mouse PD-1 antibody, RMP1-14, HY-P99144, MCE), BALB/c mice (6-8 weeks) were subcutaneously injected with CT26 cells ($1 \times 10^6$). When the tumor size reached 50-100 $mm^3$, the CT26-bearing mice were randomly divided into two groups (n=5), and injected with PBS or anti-PD1 (3.0 mg/kg) via tail-vein for a total of 4 times administered every 2 days. The tumors size was measured using a vernier caliper every other day from day 0 to day 10, and the tumor size (V) was calculated according to $V=a\times b^2/2$, in which, a is the longest diameter and b is shortest diameter of the tumor. In accordance with the animal ethics guidelines, the mice exhibiting significant signs of impaired health or the tumors size exceeding 1500 $mm^3$ were sacrificed. The tumor tissues were collected and fixed in 4% paraformaldehyde, and dehydrated in a gradient concentration of alcohol. After embedding in paraffin, the tumor tissues were sectioned and stained with hematoxylin-Eosin (H&E) according to the manufacturer's instructions. Additionally, the immunofluorescence analysis was performed following the aforementioned protocols.

**Statistical analysis.** All the data are presented as the mean ± standard deviation (s.d.) ($n \geqslant 3$). One-way ANOVA or Student's t test was performed on the experimental data by the GraphPad Prism 8 software, and the significance was indicated by asterisks (ns: not significant, $p > 0.05$, *$p < 0.05$, **$p < 0.01$, ***$p < 0.001$, ****$p < 0.0001$). Survival curves were generated using Kaplan-Meier estimates and tested using the log-rank test.

## Supplementary Figures

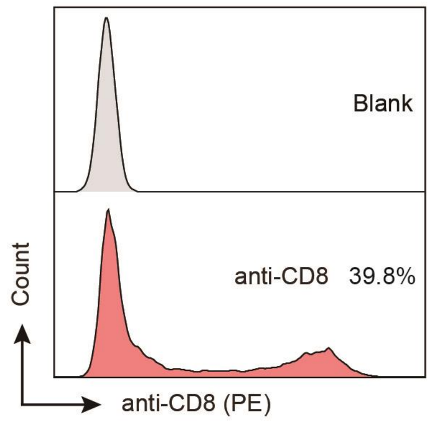


**Figure S1.** The representative FCM profiles of $CD8^+$ T cells incubated with anti-CD8 for 30 min (gated by PE labeled anti-CD8).

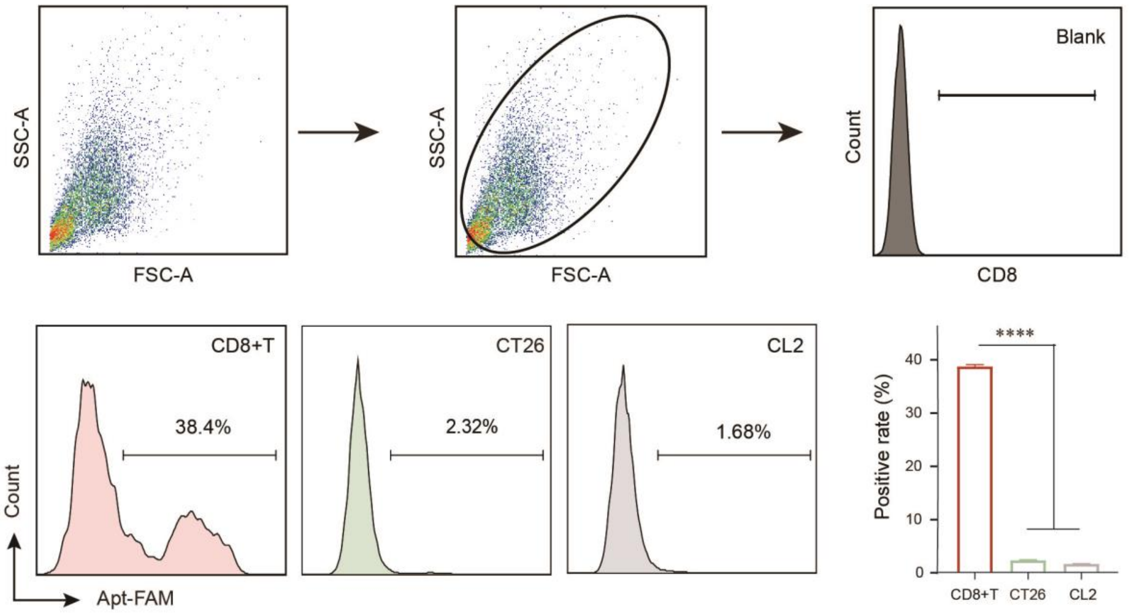


**Figure S2**. The representative FCM profiles of Apt-CD8 incubated with $CD8^+$ T cells, CT26 and CL2 cells for 30 min (gated by the FAM labeled Apt-CD8).

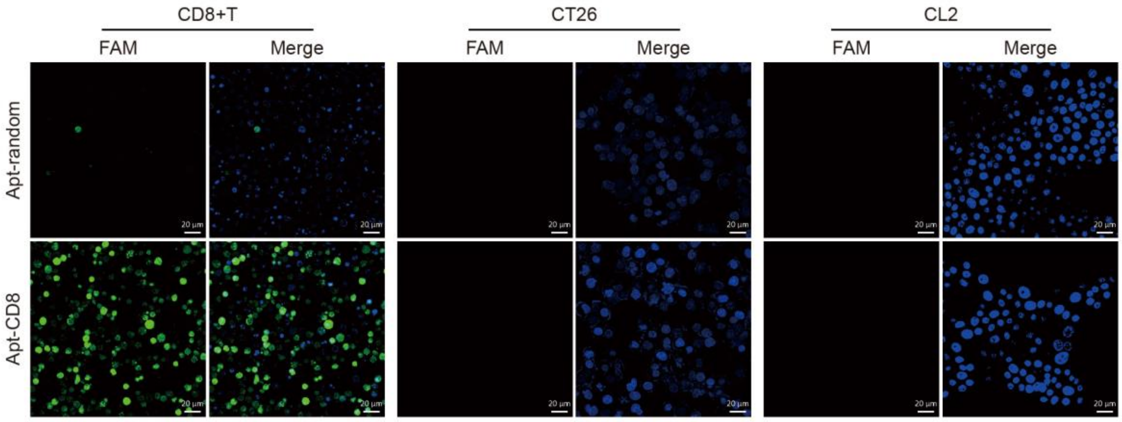


**Figure S3**. The representative confocal images of Apt-CD8 and Apt-Random incubated with $CD8^+$ T cells, CT26 and CL2 cells for 30 min (gated by the FAM labeled aptamers).

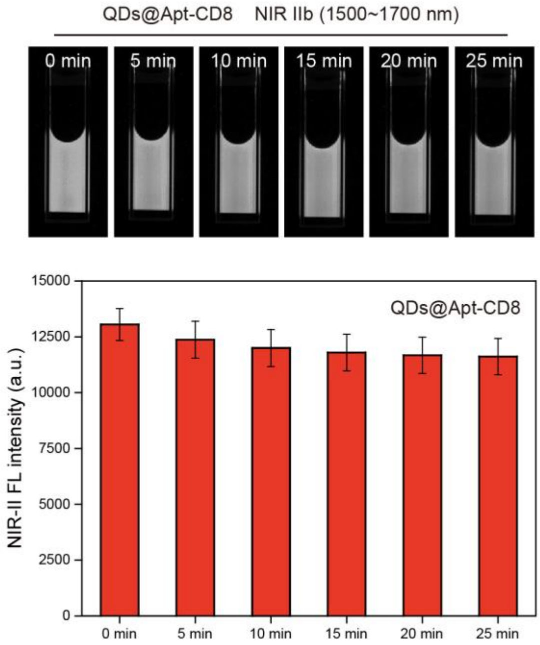


**Figure S4.** The photo-stability of QDs@Apt-CD8. NIR-II FL intensity of QDs@Apt-CD8 under continuous 808 nm laser irradiation over time with 1500 nm LP plus 1700 nm SP emission filters.

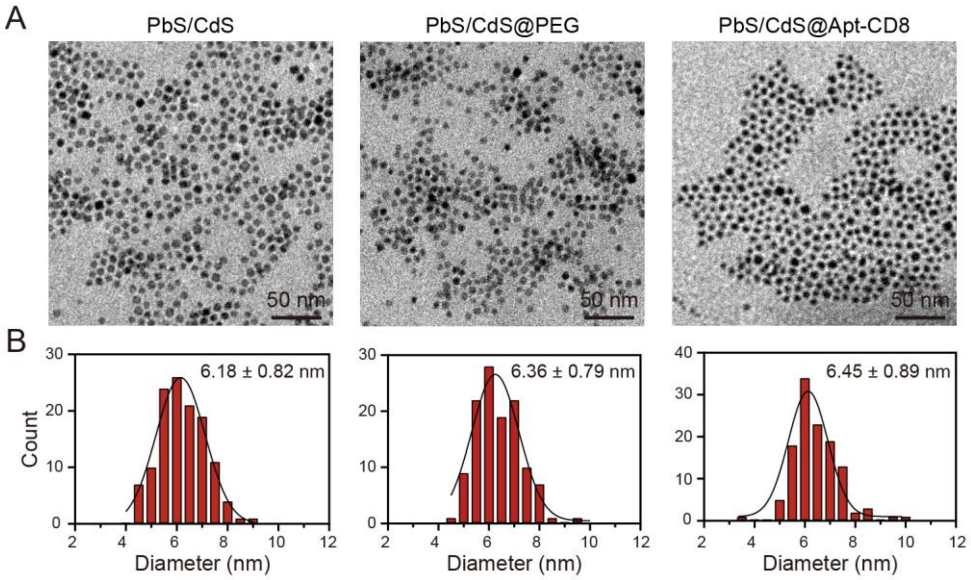


**Figure S5**. The representative TEM image and the size distribution of PbS/CdS QDs, PbS/CdS@PEG and PbS/CdS@Apt-CD8 (n=120).

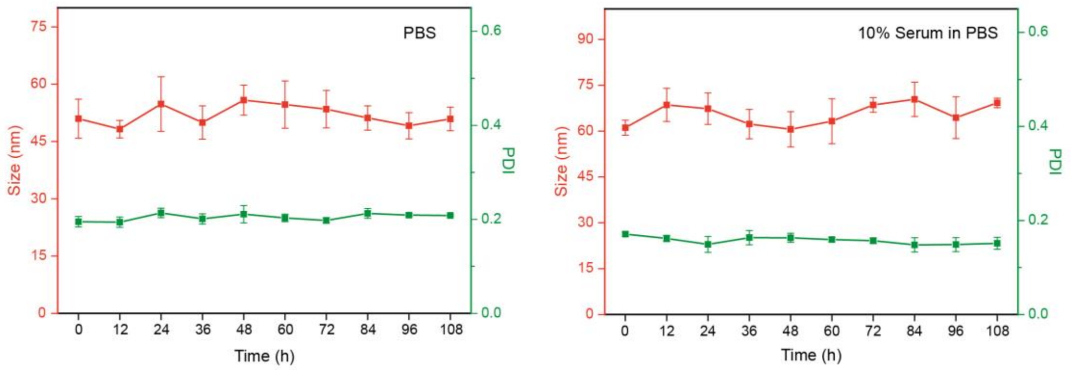


**Figure S6.** Hydrodynamic size and PDI of QDs@Apt-CD8 in physiological environments for 108 h. Data are presented as mean ± S.D (n = 3).

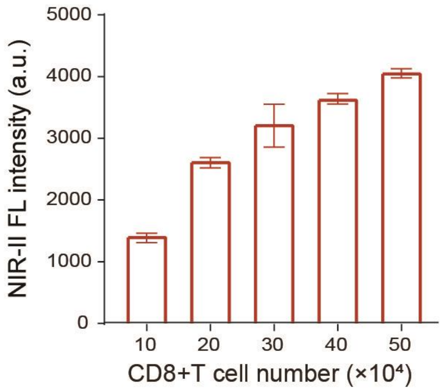


**Figure S7**. The mean fluorescence intensity of QDs@Apt-CD8 by *in vitro* NIR-II fluorescence imaging after incubation with different numbers of $CD8^{+}$ T cells for 30 min (n=3).

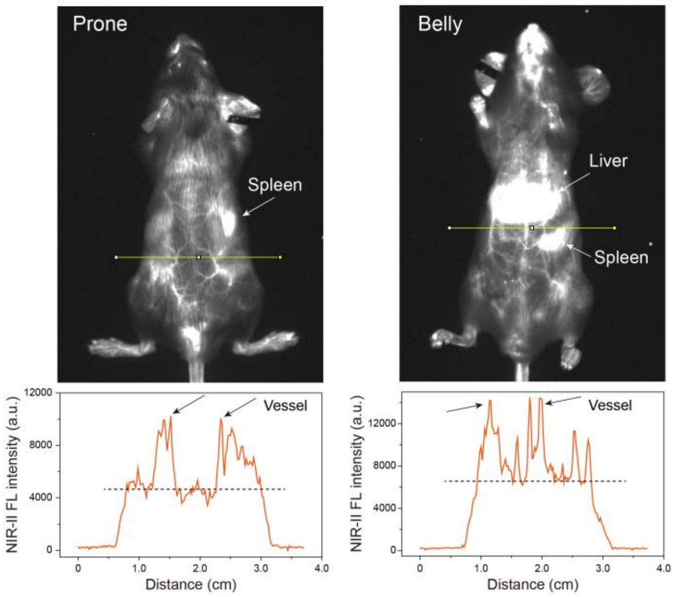


**Figure S8.** The high-contrast vessel imaging of the BALB/c mice injected with QDs@Apt-CD8 at the NIR-IIb (1500-1700 nm) window.

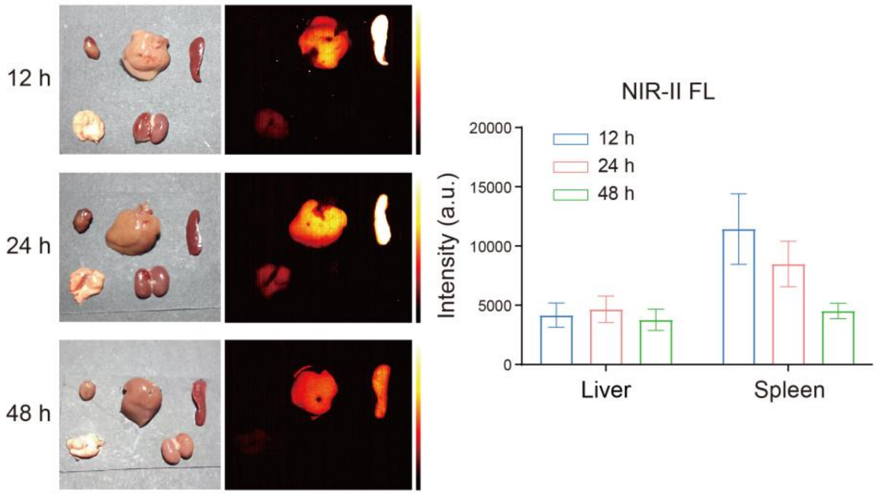


**Figure S9**. *Ex vivo* fluorescence images of mouse major organs and the mean fluorescence intensity in the liver and spleen after tail vein injection of QDs@Apt-CD8 at 12 h, 24 h and 48 h.

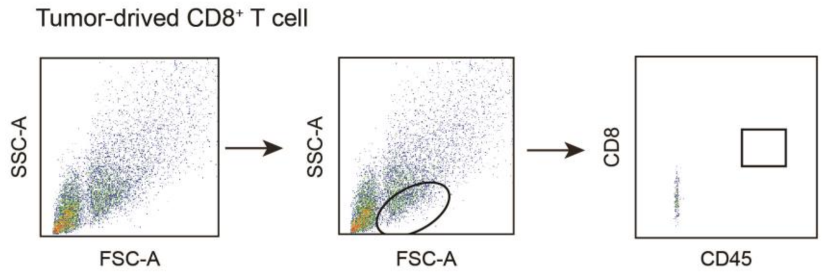


**Figure S10**. FACS gating strategy for the tumor-derived CD8$^+$ T cells in Fig. 4C.

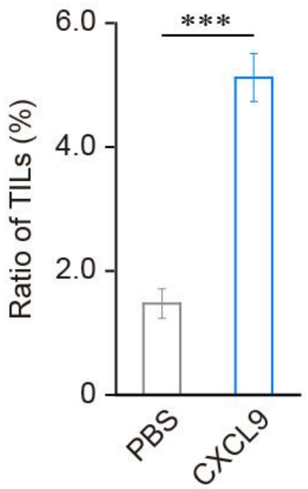


**Figure S11.** The statistical analysis of CD8$^+$ T cells from tumor regions of the mice after receiving the treatment as indicated for 12 h.

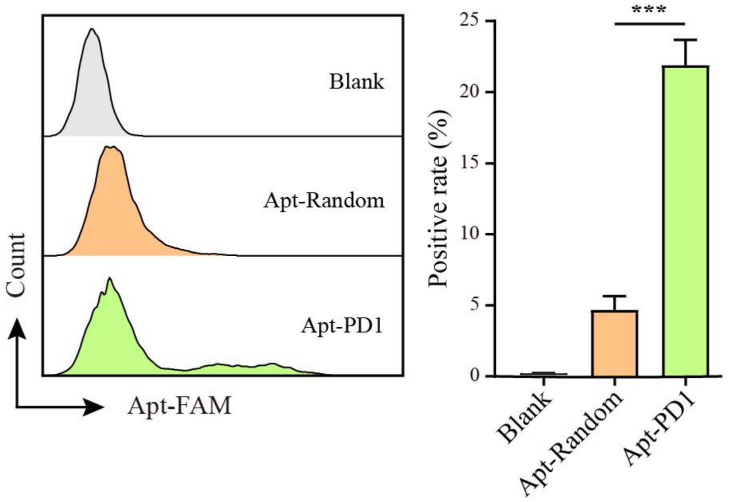


**Figure S12**. The representative FCM profiles of CD8$^+$ T cells incubated with Apt-PD1 or Apt-Random for 30 min (gated by FAM labeled aptamers)

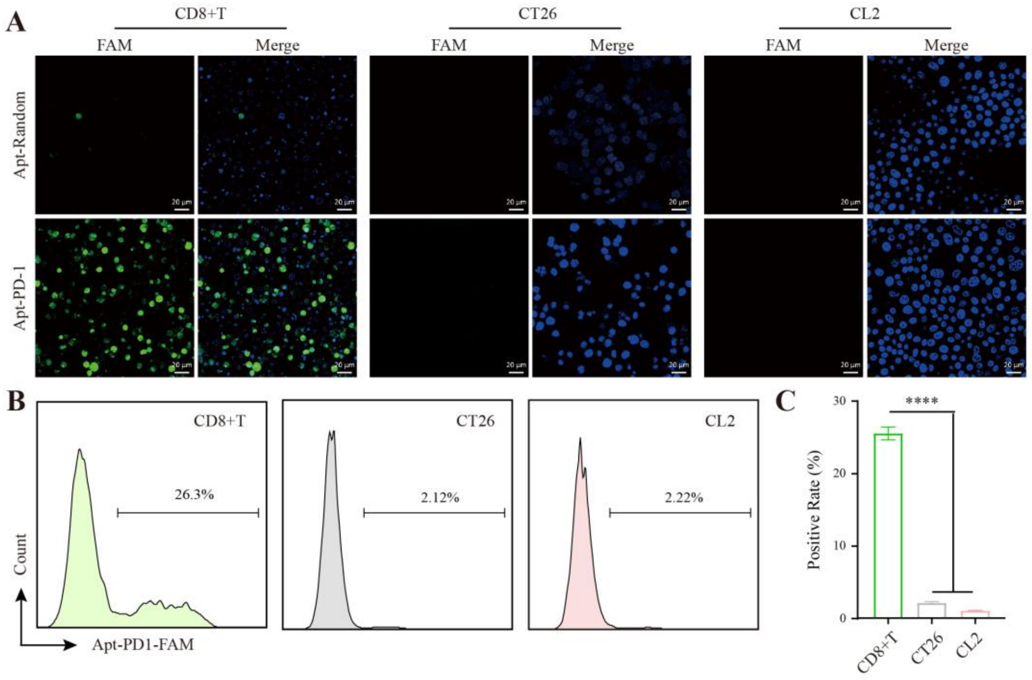


**Figure S13.** (A) The representative confocal images of Apt-CD8 and Apt-Random incubated with CD8$^{+}$ T cells, CT26 and CL2 cells for 30 min (labeled by FAM dye). (B) The representative FCM profiles of Apt-CD8 incubated with CD8$^{+}$ T cells, CT26 and CL2 cells for 30 min (gated by the FAM labeled Apt-CD8).

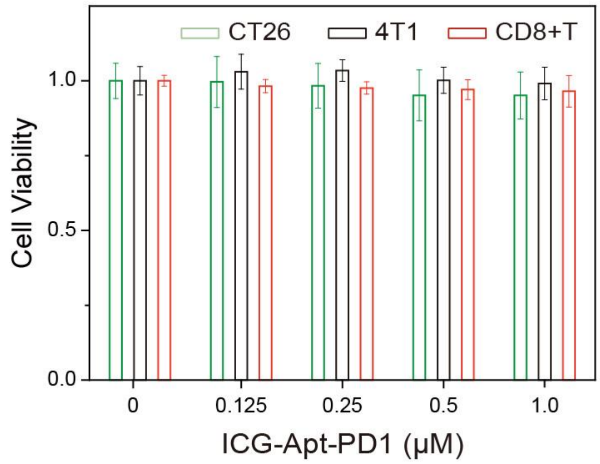


**Figure S14.** The cell viability of CT26, 4T1 and CD8$^{+}$ T cells treated with various doses of ICG-Apt-PD1 after 24 hours incubation, respectively (n = 4).

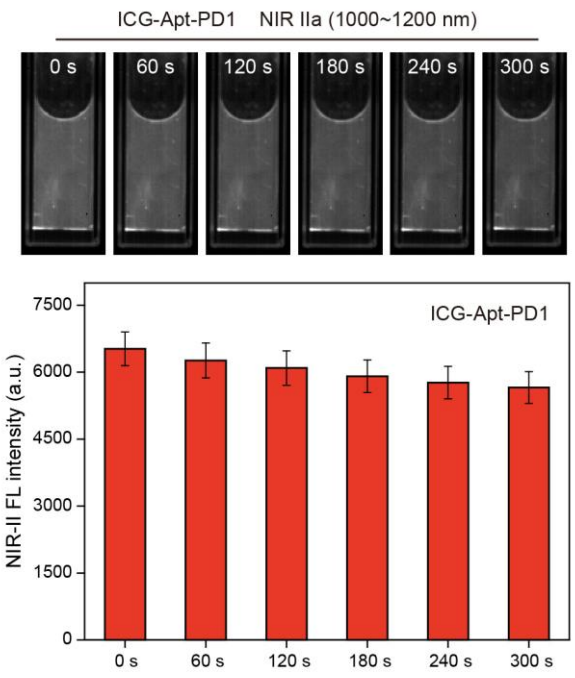


**Figure S15.** The photo-stability of ICG-Apt-PD1. NIR-II FL intensity of ICG-Apt-PD1 under continuous 808 nm laser irradiation over time with 1000 nm LP plus 1200 nm SP emission filters.

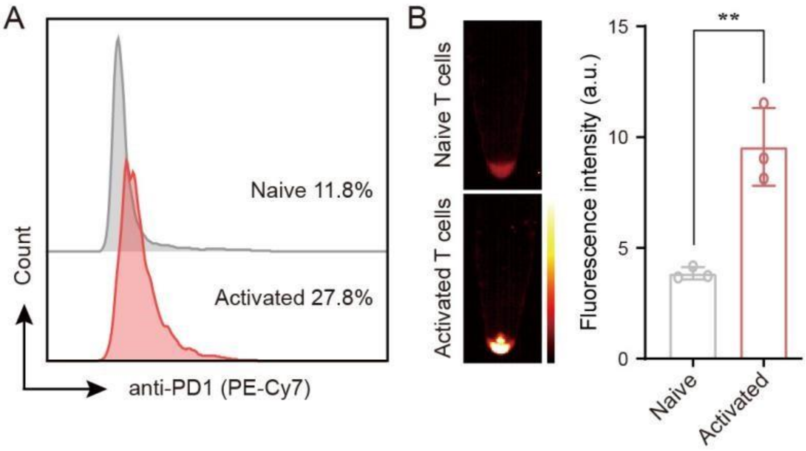


**Figure S16.** (A) The representative FCM profiles of naive or activated T cells (gated by the PE-Cy7 labeled anti-PD1). (B) *In vitro* NIR-II fluorescence images and the mean fluorescence intensity of naive or activated T cells after 30 min incubation with ICG-Apt-PD1.

To validate the ability of ICG-Apt-PD1 to target PD1, we used ICG-Apt-PD1 to evaluate the expression level of PD1 on the surface of T cells. As illustrated in Figure S16, flow cytometry results indicated that the PD1 positivity rate in activated T cells (27.8%) was 2.35 times higher than that in naive T cells (11.8%). As expected, NIR-II fluorescence imaging demonstrated a significant enhancement in the fluorescence intensity of activated T cells, approximately 2.48 times that of the naive T cells, which is consistent with the results from flow cytometry. These results suggest that ICG-Apt-PD1 has the ability to target PD1.

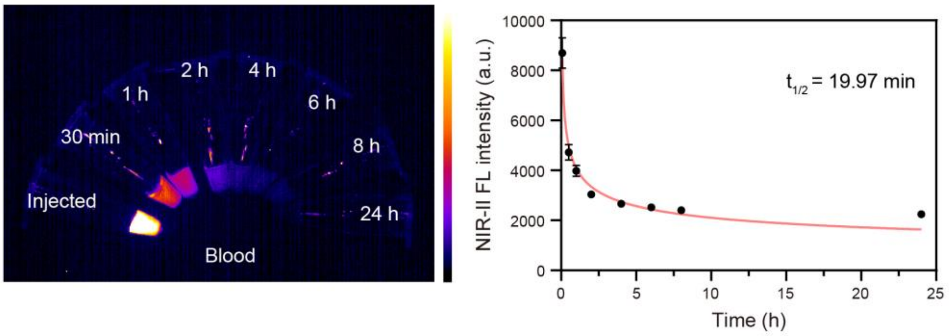


**Figure S17.** NIR-II fluorescence images and fluorescence decay curves of mouse serum after ICG-Apt-PD1 was injected via tail vein for 5 min, 30 min, 1 h, 2 h, 4 h, 6 h, 8 h and 24 h. (n=3)

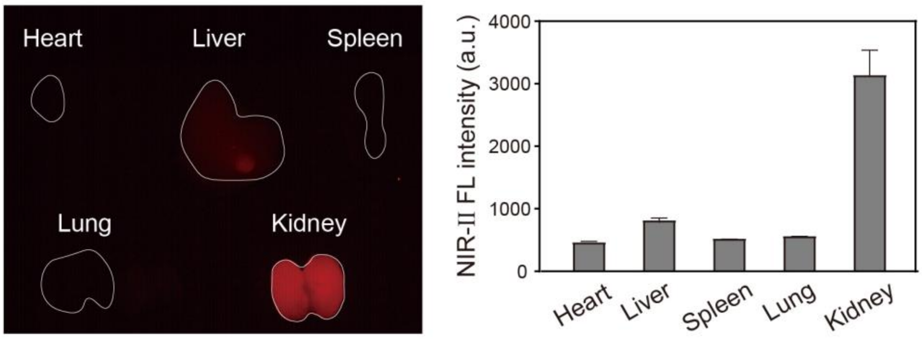


**Figure S18.** *Ex vivo* NIR-II fluorescence imaging and the intensity statistics of major organs of mice 48 h after tail vein injection of ICG-Apt-PD1.

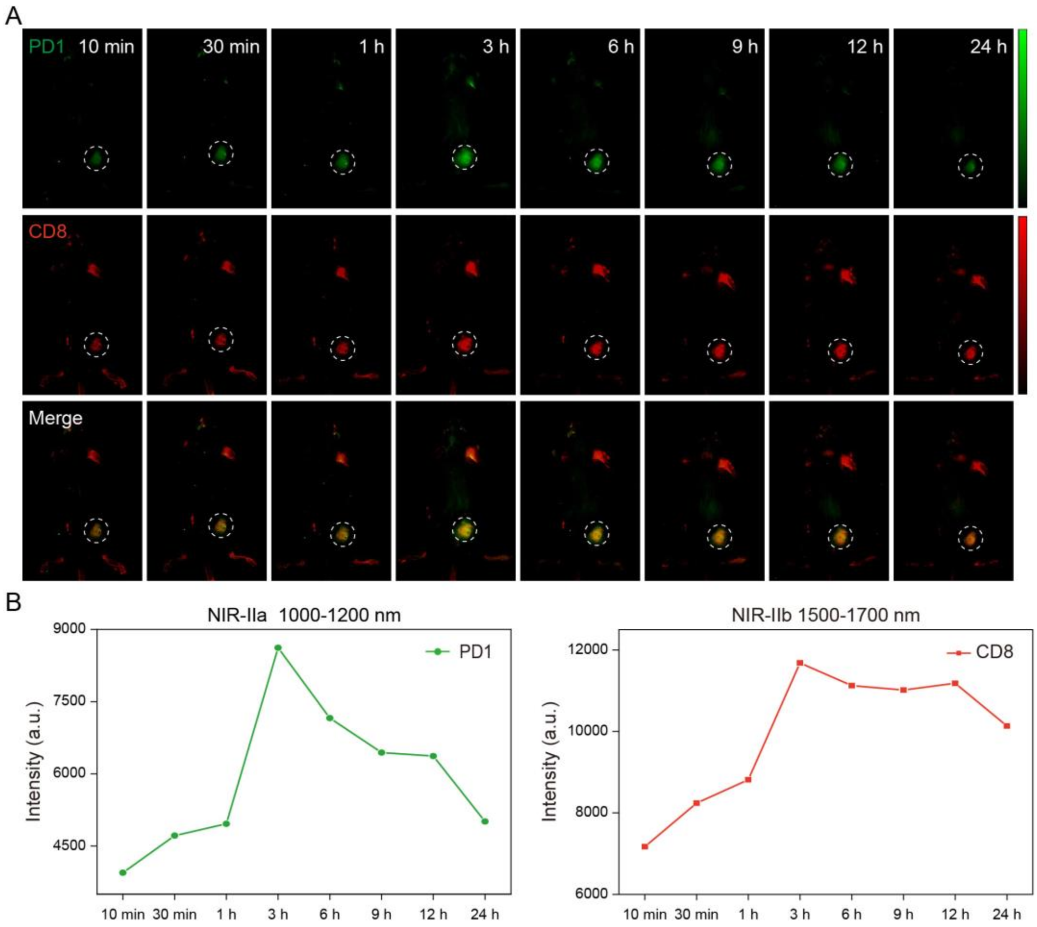


**Figure S19.** *In vivo* NIR-II two-plex fluorescence imaging of H22 tumor-bearing mice. The mice were intravenously injected with QDs@Apt-CD8 and ICG-Apt-PD1, and were imaged at different time points with 808 nm excitation.

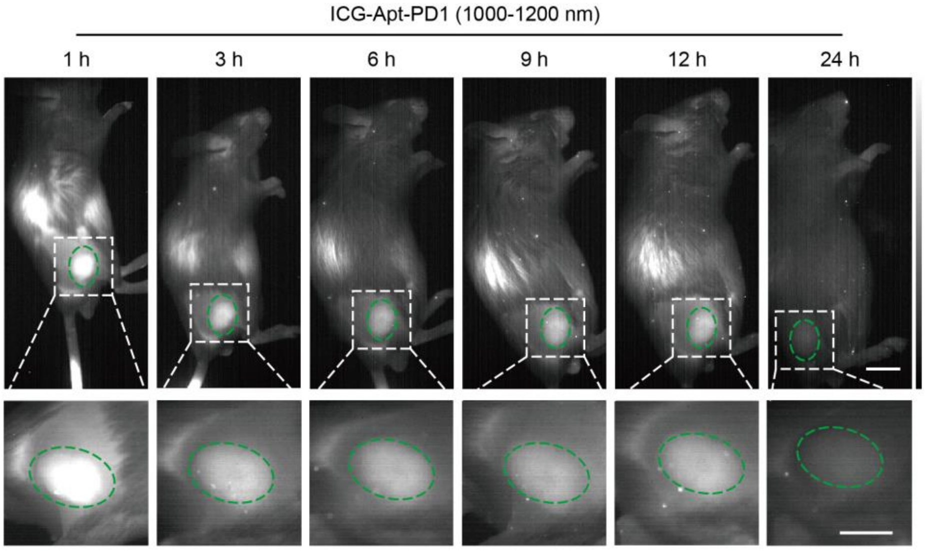


**Figure S20**. *In vivo* fluorescence imaging of CT26 tumor-bearing mouse in the NIR-IIa window. Bottom: the high magnification image for tumor region. Tumor areas were marked with green circles. The mouse was intravenously injected with ICG-Apt-PD1 and was imaged at different time points with 808 nm excitation. Scale bar, 5 mm.

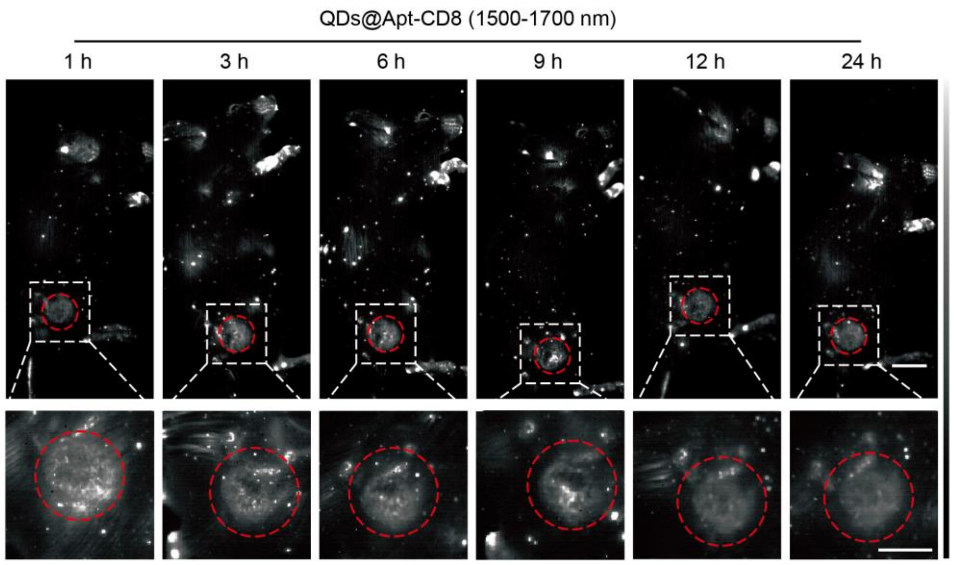


**Figure S21**. *In vivo* fluorescence imaging of CT26 tumor-bearing mouse in the NIR-IIb window. Bottom: the high magnification image for tumor region. Tumor areas were marked with red circles. The mouse was intravenously injected with QDs@Apt-CD8 and was imaged at different time points with 808 nm excitation. Scale bar, 5 mm.

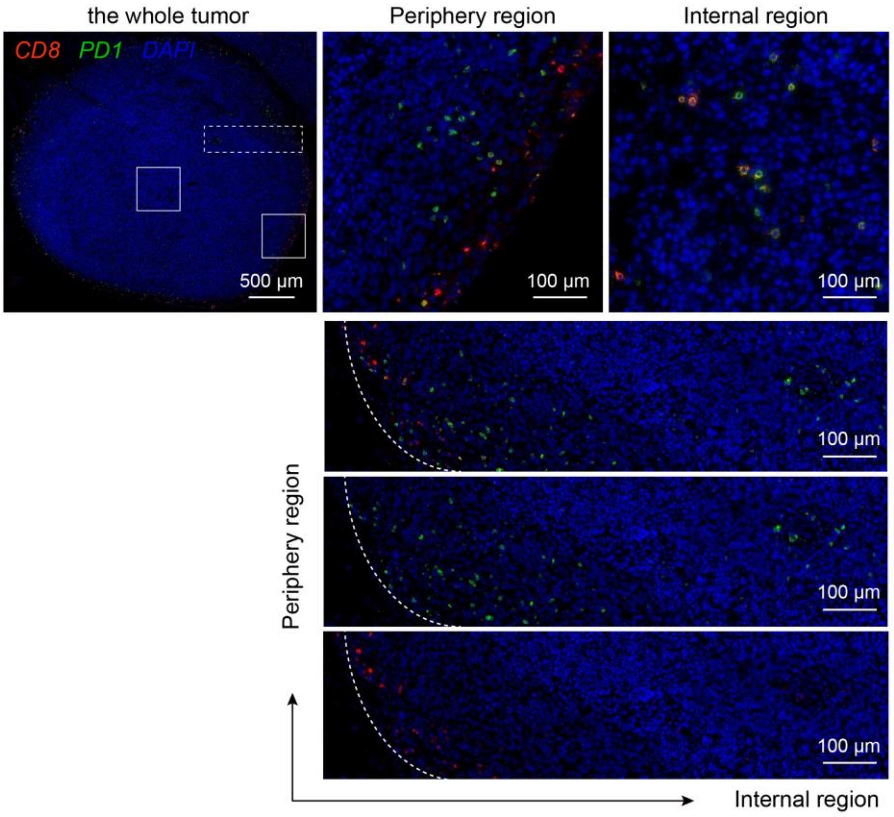


**Figure S22**. The *ex vivo* immunofluorescence images of CT26 tumor-bearing mouse after the intravenous injection of QDs@Apt-CD8 and ICG-Apt-PD1 for 24 h.

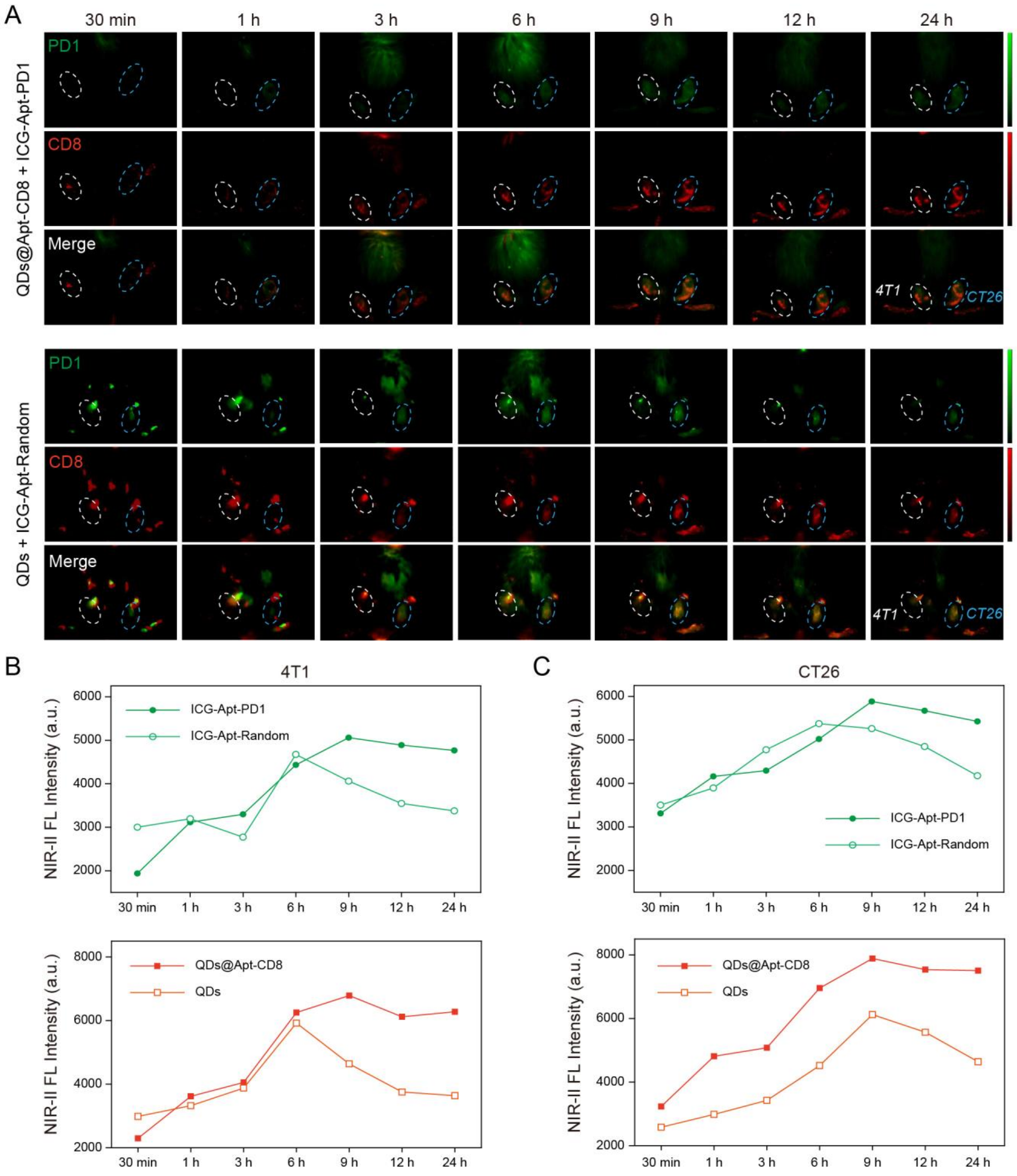


**Figure S23.** (A) *In vivo* NIR-II two-plex fluorescence imaging of 4T1/CT26 tumor-bearing mice, and the corresponding fluorescence intensity of 4T1 tumor (B) or CT26 tumor regions (C). The mice were intravenously injected with QDs@Apt-CD8 + ICG-Apt-PD1, or QDs + ICG-Apt-Random, respectively. The NIR-II images were recorded at different time points with 808 nm excitation and NIR-IIa (1000-1200 nm) and NIR-IIb (1500-1700 nm) detection range.

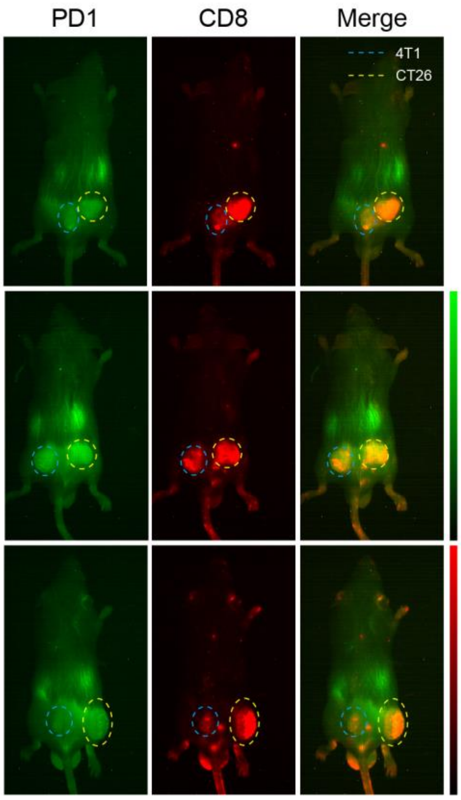


**Figure S24**. *In vivo* NIR-II multi-channel fluorescence imaging of tumor regions from bilateral tumor-bearing mouse (Left, 4T1 tumor model; Right, CT26 tumor model) after the intravenous injection of QDs@Apt-CD8 and ICG-Apt-PD1 for 12 h. (n=3).

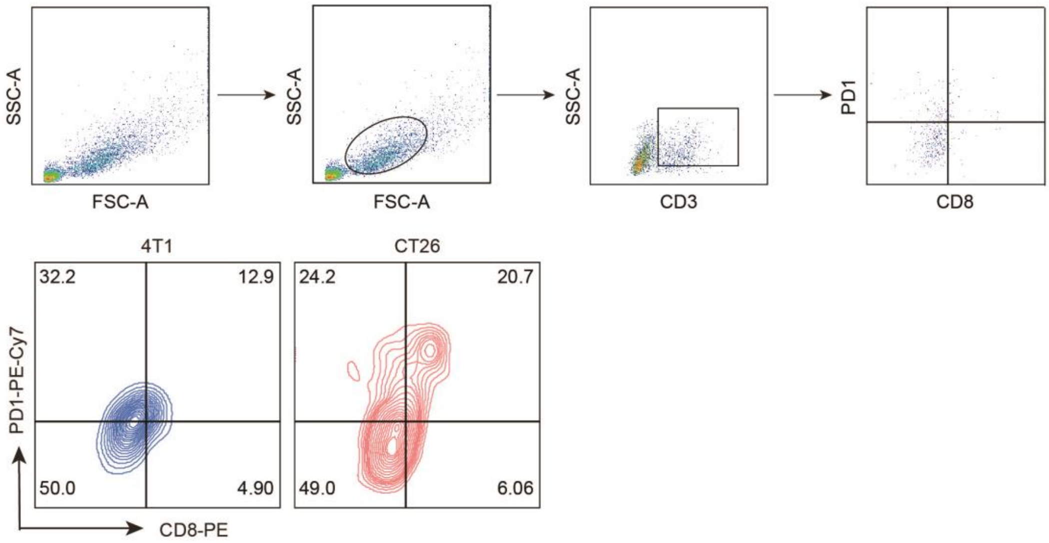


**Figure S25**. Flow cytometry profiles of PD1$^+$ T and CD8$^+$ T cells in tumor of 4T1/CT26 tumor-bearing mouse (gated by PE labeled anti-CD8 and PE-Cy7 labeled anti-PD1). (n=3)

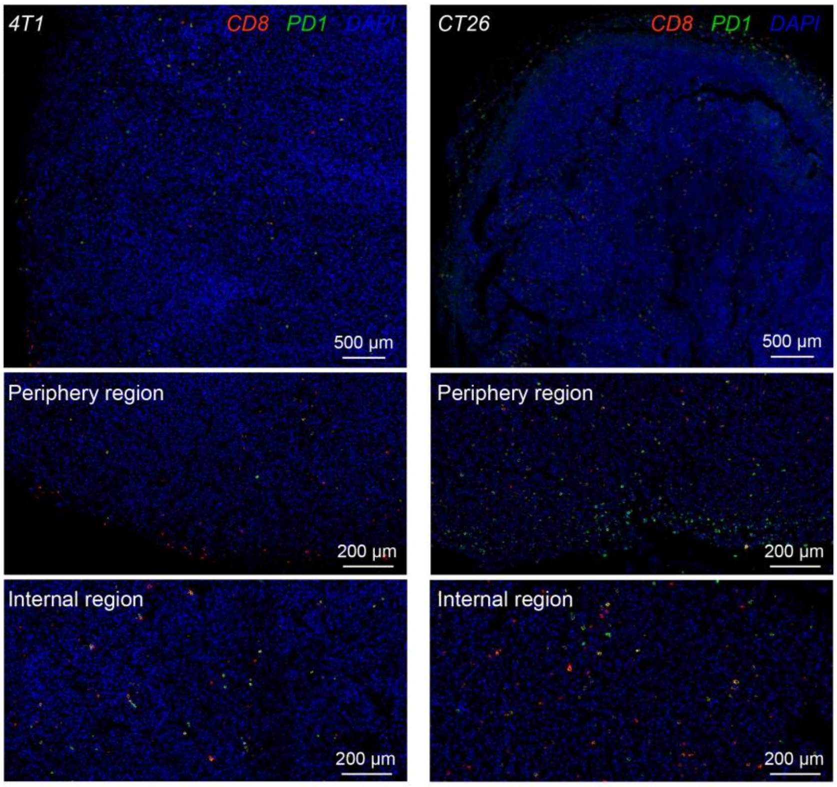


**Figure S26**. *Ex vivo* immunofluorescence images of tumor regions from bilateral tumor-bearing mouse. Left, 4T1 tumor model; Right, CT26 tumor model.

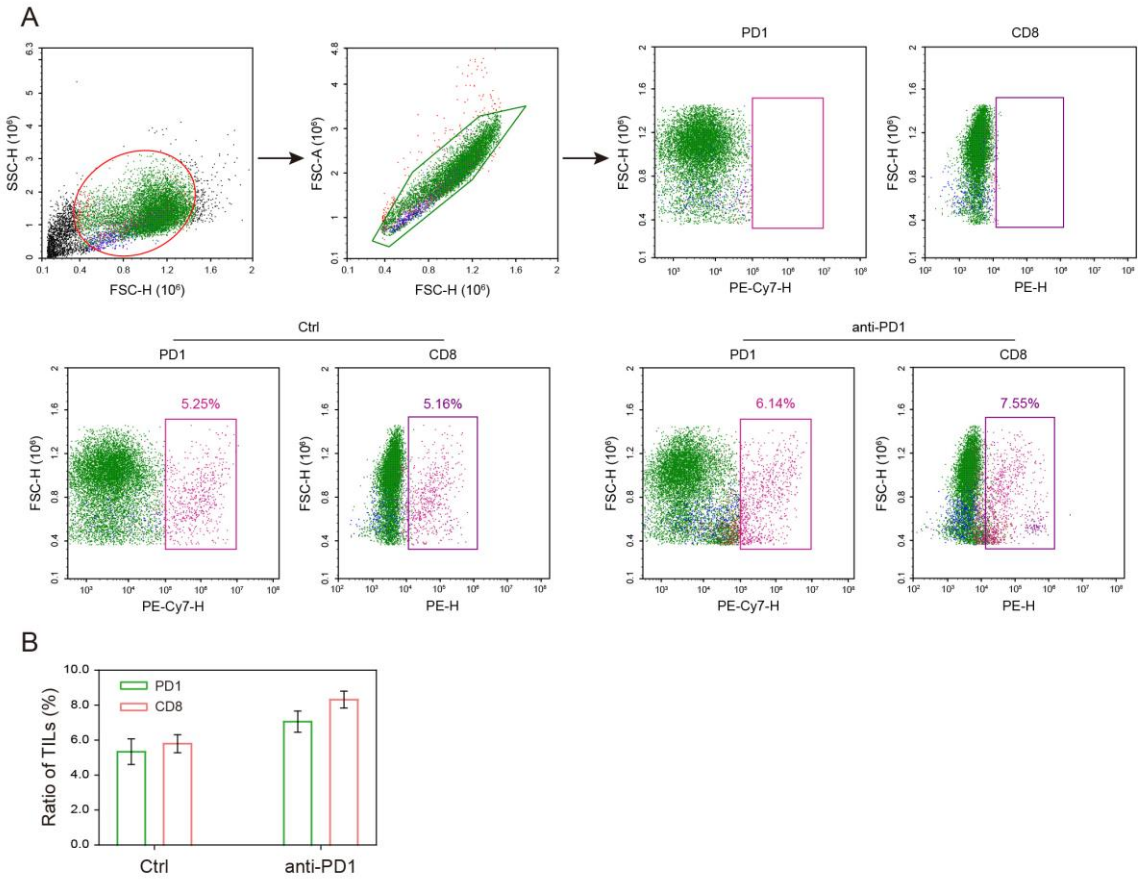


**Figure S27.** Flow cytometry profiles and the statistical analysis of $CD8^+$ T cells and PD1 in tumor from the mice treated with anti-PD1 or PBS.

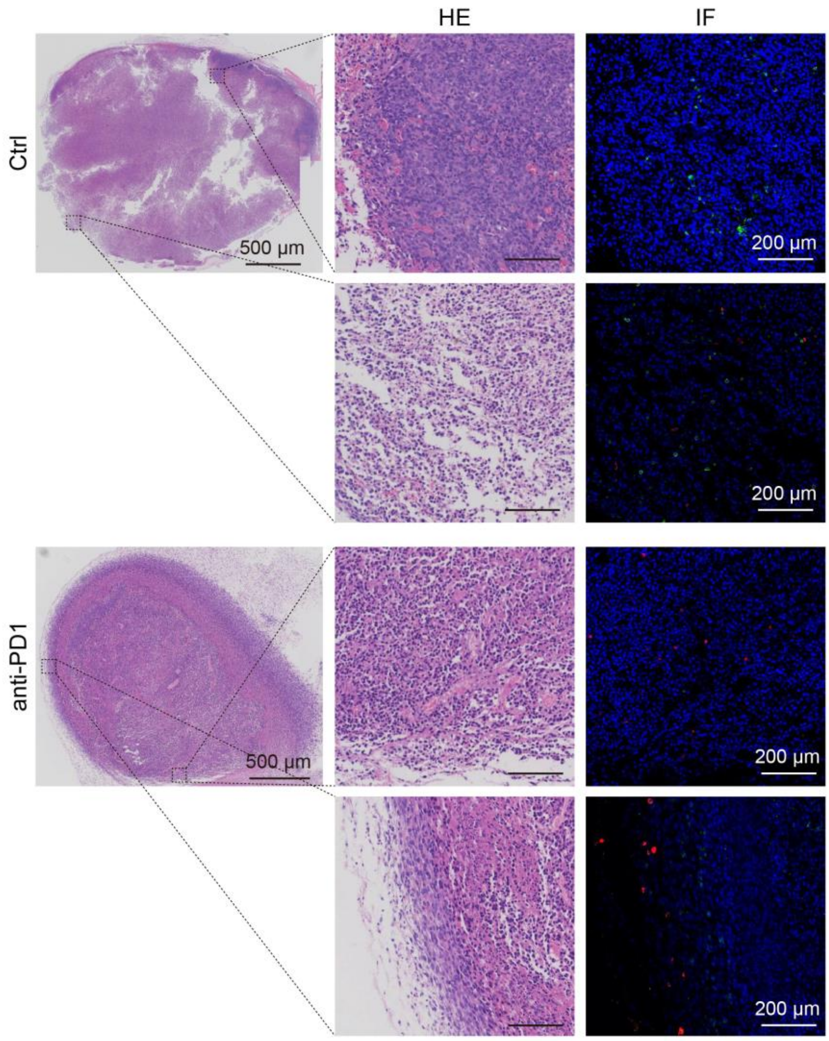


**Figure S28**. Hematoxylin-eosin (HE) staining and *ex vivo* immunofluorescence images of tumor regions from CT26 tumor-bearing BALB/c mice after receiving different treatments as indicated.

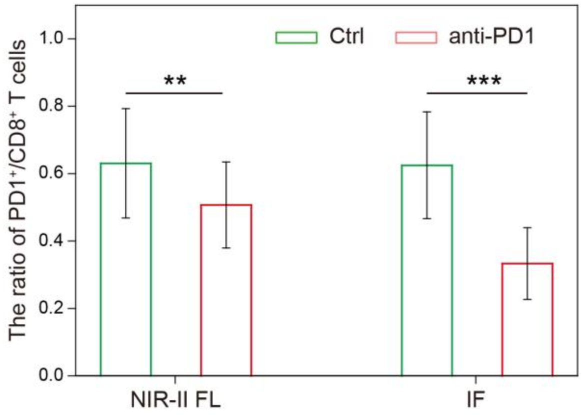


**Figure S29**. The ratio of $PD1^+$ T cells and $CD8^+$ T cells in tumor of CT26 tumor-bearing BALB/c mice after receiving anti-PD1 treatment, detected by NIR-II FL or IF. (**p < 0.01, ***p < 0.001, n=3, one-way ANOVA followed by Tukey`s multiple comparison test.)

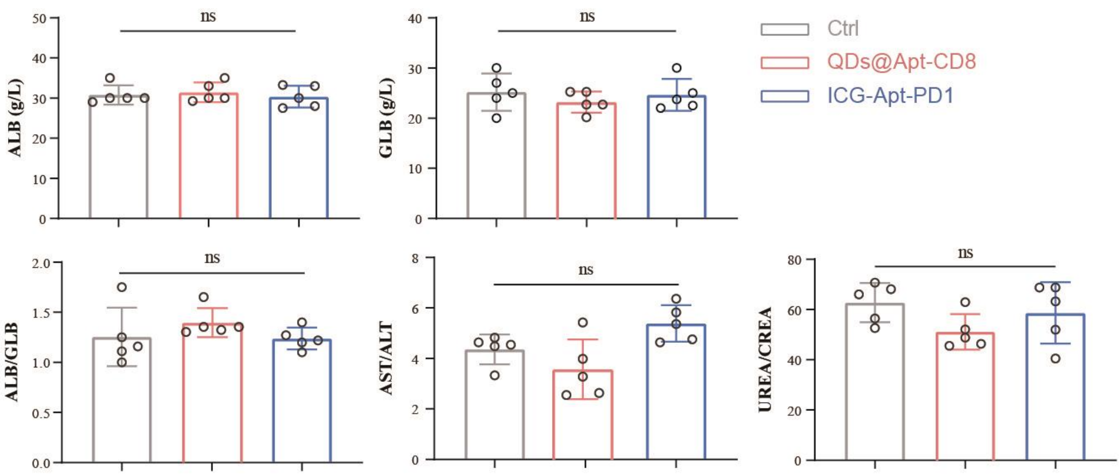


**Figure S30**. Biosafety assessment of the QDs@Apt-CD8 and ICG-Apt-PD1 probe. Blood biochemical and cytokines test of mice after indicated treatments. ns represents not significant, Data are presented as mean ± S.D. (n = 5, biologically independent replicates). Abbreviations: ALB, Albumin; GLB, Globulin; AST, aspartate aminotransferase; ALT, alanine aminotransferase; ALP, alkaline phosphatase; CREA, creatinine; UREA, urea.

## Supplementary Tables

**Table S1.** The sequences of oligonucleotides for aptamers

| **Name** | **Sequences (5`-3`)** |
|---|---|
| Apt-CD8-FAM | 5`-FAM-TTTTTTTTTTCCAGAGTGACGCAGCAACAGAGGTGTAGAAG TACACGTGAACAAGCTTGAAATTGTCTCCGACAGAGGTGGACACGGT GGCTTTTAGT-3` |
| Apt-PD1-FAM | 5`-FAM-GACGATAGCGGTGACGGCACAGACGGTACAGTTCCCGTCCC TGCACTACACGTATGCCGCTTCCGTCCGTCGCTC-3` |
| Apt-random-FAM | 5`-FAM-TTTTTAAGTAATGTATTTAAATTGCGAACTACTACTCTCTCT ATATATTGCGAACTACTACTCTCTCTATATTAAAGGAAGGATCTCA-3` |
| Apt-CD8-SH | 5`-CCAGAGTGACGCAGCAACAGAGGTGTAGAAGTACACGTGAACAAG CTTGAAATTGTCTCCGACAGAGGTGGACACGGTGGCTTTTAGT-3`-SH |
| ICG-Apt-PD1 | 5`-ICG-GACGATAGCGGTGACGGCACAGACGGTACAGTTCCCGTCCCT GCACTACACGTATGCCGCTTCCGTCCGTCGCTC-3` |
